# TOP2DFVT: An Efficient Matlab Implementation for Topology Optimization based on the Finite-Volume Theory

**DOI:** 10.12688/f1000research.150945.1

**Published:** 2024-07-16

**Authors:** Marcelo Araujo, Arnaldo Santos Júnior, Romildo Escarpini Filho, Eduardo Lages, Marcio Cavalcante

**Affiliations:** 1 Universidade Federal de Alagoas, Maceió, State of Alagoas, Brazil

**Keywords:** topology optimization, compliance minimization problem, finite-volume theory, Matlab.

## Abstract

The finite-volume theory has shown to be numerically efficient and stable for topology optimization of continuum elastic structures. The significant features of this numerical technique are the local satisfaction of equilibrium equations and the employment of compatibility conditions along edges in a surface-averaged sense. These are essential properties to adequately mitigate some numerical instabilities in the gradient version of topology optimization algorithms, such as checkerboard, mesh dependence, and local minima issues. Several computational tools have been proposed for topology optimization employing analysis domains discretized with essential features for finite-element approaches. However, this is the first contribution to offer a platform to generate optimized topologies by employing a Matlab code based on the finite-volume theory for compliance minimization problems. The Top2DFVT provides a platform to perform 2D topology optimization of structures in Matlab, from domain initialization for structured meshes to data post-processing. This contribution represents a significant advancement over earlier publications on topology optimization based on the finite-volume theory, which needed more efficient computational tools. Moreover, the Top2DFVT algorithm incorporates SIMP and RAMP material interpolation schemes alongside sensitivity and density filtering techniques, culminating in a notably enhanced optimization tool. The application of this algorithm to various illustrative cases confirms its efficacy and underscores its potential for advancing the field of structural optimization.

## 1. Introduction

In structural engineering, topology optimization is a technique that searches for the best material distribution inside an analysis domain based on an objective function and one or more constraints ( Bendsøe and Sigmund, 2003). Therefore, topology optimization allows for the discovery of innovative and high-performance structural designs, which attracted the interest of mathematicians and engineers ( Liu and Tovar, 2014). With the progressive development of computer technology and computational mechanics over the last decades, the structural topology optimization tools have gradually experienced improvements that allow the solution of medium and large-scale problems. In addition, topology optimization has become an effective strategy for generating innovative forms for additive manufacturing, architectural design, and engineering ( Zhuang et al., 2023). In general, compliance evaluation has played an important role in topology optimization algorithms. Since the pioneer work of Michell (1904), who derived the optimality criteria (OC) method, and the reconstruction proposed by Bendsøe and Kikuchi (1988), a great part of the advances in topology optimization has been achieved by employing methodologies based on structural compliance minimization problems. Some studies on this field can still be found in Liu et al. (2023), Yi et al. (2023), Lee et al. (2023), Arruda et al. (2022), Bouajila et al. (2021), and Ferrari and Sigmund (2020).

In topology optimization algorithms, the interest is in determining whether we should put material or not, which generates a “black and white” design. Therefore, the structural material distribution is obtained by a binary “0-1”, where 0 indicates void and 1 indicates the presence of material. However, this kind of topology optimization algorithms lead to an integer programming problem, which has revealed to be an unfeasible approach for large scale topology optimization problems. An alternative approach is the SIMP ( *Solid Isotropic Material with Penalization*) method, which has been extensively used due to its versatility, convergence, and ease implementation ( Rozvany, 2009). In this approach, the material properties can be evaluated inside each element of the discretized domain, and the design variables are the elements’ relative densities. Therefore, the mechanical properties are modeled by the material relative density raised to a penalty factor that penalizes their intermediate values. Another interpolation scheme to penalize intermediate values of relative density is the RAMP ( *Rotational Approximation of Material Properties*) method proposed by Stolpe and Svanberg (2001), which employs a concave penalty function to suppress these intermediate values in the objective function. Unlike the SIMP method, the RAMP model presents non-zero sensitivity at zero density, so this model is especially efficient to remedy some numerical difficulties presented in problems with very low densities ( Deaton and Grandhi, 2014).

Different authors have developed educational algorithms to design optimized topologies in the last two decades. The trailblazer top99 educational code written in Matlab proposed by Sigmund (2001) had promoted important impacts in the topology optimization field, such as teaching of topology optimization tools in undergraduate courses, building simple code for new researchers, and pioneering a new popular category of publications in the structural optimization field: educational articles self-containing compact codes for teaching and research ( Zhou and Sigmund, 2021). Beyond the well-known top99 Matlab code, several computer tools for Matlab and other platforms are available, such as PETSc by Smit et al. (2021) and Aage et al. (2015) for Python; TopOpt app by Aage et al. (2013) for language C; Stutz et al. (2022), Aage and Lazarov (2013), and Borrvall and Petersson (2001) for C++ language; Liu et al. (2005) for Femlab; and Sokół (2011) for Mathematica. However, a significant part of the proposed educational algorithms for topology optimization is written in Matlab language, as top99neo by Ferrari and Sigmund (2020), an 88-line code for parametrized level-set method by Wei et al. (2018), top88 by Andreassen et al. (2011), top3d by Liu and Tovar (2014), PolyTop by Talischi et al. (2012), HoneyTop90 by Kumar (2023), a 115-line code for multi-material topology optimization by Tavakoli and Mohseni (2014), and GRAND by Zegard and Paulino (2014).

In the top99 topology optimization code, the performance of several operations can be increased by exploiting the strengths of Matlab, such as loop vectorization and memory preallocation, and by restructuring the program, as moving portions of code out of the optimization loop so they would be executed once ( Andreassen et al., 2011). Therefore, Andreassen et al. (2011) have proposed an 88-line code in Matlab for compliance minimization by allocating these computational features (top88), which has substantially improved the computational performance of the optimization algorithm. Later, Liu and Tovar (2014) have extended this algorithm to three-dimensional problems by also placing other strategies for topology optimization of compliant mechanisms and heat conduction problems. With the evolution of topology optimization research field and Matlab, the top88 code has become outdated, which has motivated the publication of the new generation of the top99 code (top99neo) by Ferrari and Sigmund (2020), making some improvements inmet the assembly operations, accelerating the Optimality Criteria (OC) method, filters implementation, and extending to three-dimensional structures.

Araujo et al. (2020a, b) propose applying the finite-volume theory for topology optimization considering compliance minimization. This theory has been shown to be numerically stable for optimization problems, especially its checkerboard-free property, even when a non-filtering technique is employed. Numerical stability is an essential feature of the finite-volume theory applied in topology optimization tools to obtain more reliable optimized topologies. Also, this technique has shown to be well suitable method for elastic stress analysis in solid mechanics, investigations of its numerical efficiency can be found in Araujo et al. (2021), Cavalcante et al. (2007a, b, 2008) and Cavalcante and Pindera (2012a, b). The satisfaction of equilibrium equations at the subvolume level, concomitant to kinematic and static continuities established in a surface-averaged sense between common faces of adjacent subvolumes, are features that distinguish the finite-volume theory from the finite-element method. Thus, in the finite-volume theory, the connections between adjacent subvolumes occur through subvolumes’ faces, which is more likely from the continuum mechanics point of view.

This contribution provides a new topology optimization tool for the analysis of 2D structures using the Matlab language, which starts from domain discretization and continues until data is post-processed. In addition, this is the first time a platform for optimizing structures using the finite-volume theory can be applied to medium and large-scale problems, besides obtaining checkerboard-free and mesh-independent designs. The topology optimization tool also incorporates the SIMP and RAMP methods and the sensitivity and density filters. Employing a symmetric modified stiffness matrix also represents an advance since it accelerates the algorithm and establishes a relation between resultant forces and displacements instead of tractions and displacements, which are energetically conjugated static and kinematic quantities. These improvements have dramatically reduced the computational cost and solved the oscillatory phenomenon issue through the RAMP approaches, especially compared with the results in Araujo et al. (2020a). More details about the implementation can be found in the GitHub link ( https://github.com/fvt7782/Top2DFVT).

## 2. Finite-volume theory

In general, the finite-volume theory employs the stress and displacement fields and imposes boundary and continuity conditions between adjacent subvolumes in an average-sense, which has guaranteed the checkerboard-free property discussed in Araujo et al. (2020a). Additionally, the differential equilibrium equations are locally satisfied in an average-sense ( Araujo et al., 2021), and the displacement field in the subvolume is modeled by second-order polynomials defined in local coordinates ( Cavalcante et al., 2007a). The presented formulation has its roots in the standard version of the finite-volume theory presented in Cavalcante and Pindera (2012a) for structured meshes formed by rectangular subvolumes. Fundamentally, the structural analysis problem involves mechanical quantities evaluation, as applied loads, internal forces, displacements, and strains. The main objective is determining the stress and displacements when structural discretized domains are employed, where stress-strain relation can be easily expressed.

**Figure 1.**
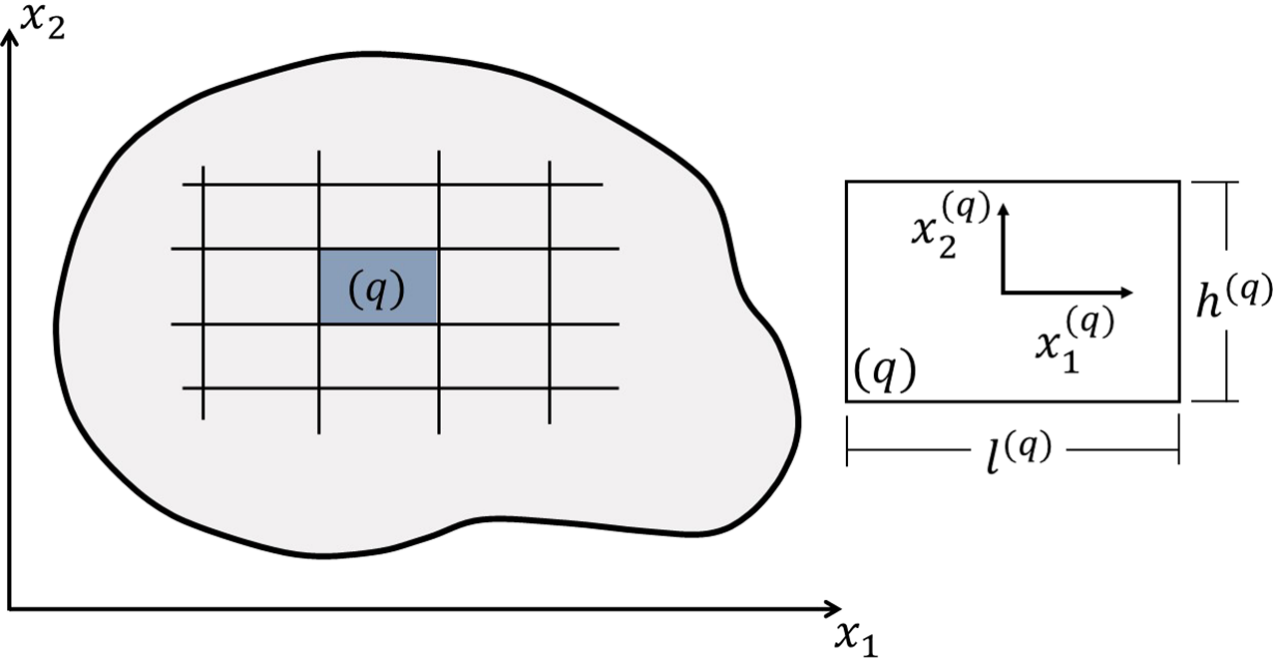
Discretized reference domain and global coordinate system (left) and subvolume and local coordinate system (right).

**Figure 2.**
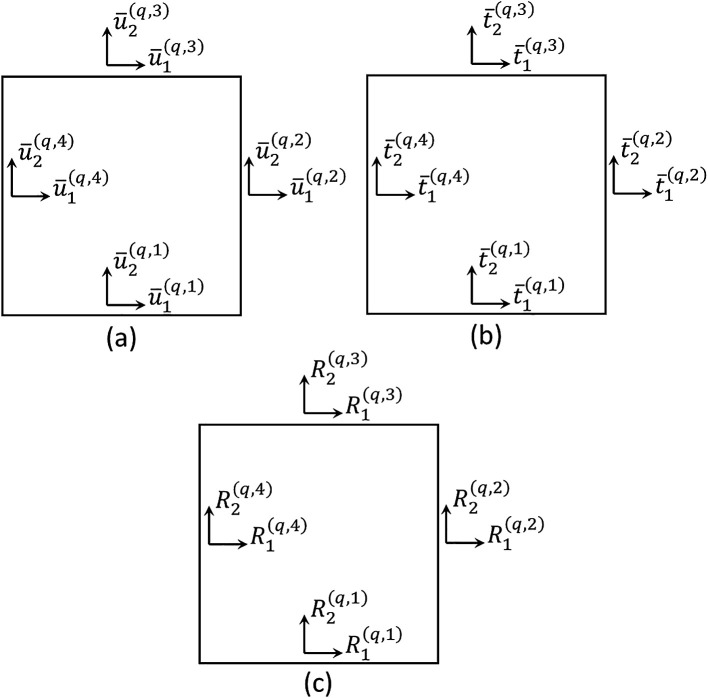
Degrees of freedom in a generic subvolume: (a) surface-averaged displacements, (b) surface-averaged tractions, and (c) resultant forces along edges.

Figure 1 presents the analysis domain in $x_{1}-x_{2}$ plane, which is discretized in $N_{q}$ subvolumes. The subvolume dimensions are $l^{\left(q\right)}$ and $h^{\left(q\right)}$ for $q=1,\ldots ,N_{q}$ , where $x_{1}^{\left(q\right)}$ and $x_{2}^{\left(q\right)}$ represent the local coordinate system. Following Cavalcante and Pindera (2012a), the displacement of a subvolume q can be approximated by an incomplete quadratic version of Legendre polynomial expansion in the local coordinate system as follows:

$$u_{i}^{\left(q\right)}=W_{i\left(00\right)}^{\left(q\right)}+x_{1}^{\left(q\right)}W_{i\left(10\right)}^{\left(q\right)}+x_{2}^{\left(q\right)}W_{i\left(01\right)}^{\left(q\right)}+\frac{1}{2}\left(3{x_{1}^{\left(q\right)}}^{2}-\frac{{l^{\left(q\right)}}^{2}}{4}\right)W_{i\left(20\right)}^{\left(q\right)}+\frac{1}{2}\left(3{x_{2}^{\left(q\right)}}^{2}-\frac{{h^{\left(q\right)}}^{2}}{4}\right)W_{i\left(02\right)}^{\left(q\right)},$$ (1)

where i=1,2 and $W_{i\left(\mathrm{mn}\right)}^{\left(q\right)}$ are unknown coefficients of the displacement field. Therefore, the surface-averaged displacement components of a generic subvolume are represented in Figure 2(a) and can be defined as

$$\begin{array}{c} {\bar{u}}_{i}^{\left(q,p\right)}=\frac{1}{l^{\left(q\right)}}\int_{-\frac{l^{\left(q\right)}}{2}}^{\frac{l^{\left(q\right)}}{2}}u_{i}\left(x_{1}^{\left(q\right)},\mp \frac{h^{\left(q\right)}}{2}\right)dx_{1}^{\left(q\right)},\text{for}\;p=1,3\;\\ {\bar{u}}_{i}^{\left(q,p\right)}=\frac{1}{h^{\left(q\right)}}\int_{-\frac{h^{\left(q\right)}}{2}}^{\frac{h^{\left(q\right)}}{2}}u_{i}\left(\pm \frac{l^{\left(q\right)}}{2},x_{2}^{\left(q\right)}\right)dx_{2}^{\left(q\right)},\text{for}\;p=2,4 \end{array}.$$ (2)

Similarly, considering the application of Cauchy’s law and the plane stress state, the surface-averaged traction components at the subvolume faces can be evaluated as

$$\begin{array}{c} {\bar{t}}_{i}^{\left(q,p\right)}=\mp \frac{1}{l^{\left(q\right)}}\int_{-\frac{l^{\left(q\right)}}{2}}^{\frac{l^{\left(q\right)}}{2}}\sigma_{2i}\left(x_{1}^{\left(q\right)},\mp \frac{h^{\left(q\right)}}{2}\right)dx_{1}^{\left(q\right)},\text{for}\;p=1,3\;\\ {\bar{t}}_{i}^{\left(q,p\right)}=\pm \frac{1}{h^{\left(q\right)}}\int_{-\frac{h^{\left(q\right)}}{2}}^{\frac{h^{\left(q\right)}}{2}}\sigma_{1i}\left(\pm \frac{l^{\left(q\right)}}{2},x_{2}^{\left(q\right)}\right)dx_{2}^{\left(q\right)},\text{for}\;p=2,4 \end{array},$$ (3)

where ${\bar{t}}_{i}^{\left(q,p\right)}$ are adequately represented in Figure 2(b).

Following Araujo et al. (2020a), the local system of equations for a generic subvolume can be established as

$${\bar{t}}^{\left(q\right)}=K^{\left(q\right)}{\bar{u}}^{\left(q\right)},$$ (4)

where ${\bar{u}}^{\left(q\right)}={\left[{\bar{u}}_{1}^{\left(q,1\right)},{\bar{u}}_{2}^{\left(q,1\right)},{\bar{u}}_{1}^{\left(q,2\right)},{\bar{u}}_{2}^{\left(q,2\right)},{\bar{u}}_{1}^{\left(q,3\right)},{\bar{u}}_{2}^{\left(q,3\right)},{\bar{u}}_{1}^{\left(q,4\right)},{\bar{u}}_{2}^{\left(q,4\right)}\right]}^{T}$ is the local surface-averaged displacement vector, ${\bar{t}}^{\left(q\right)}={\left[{\bar{t}}_{1}^{\left(q,1\right)},{\bar{t}}_{2}^{\left(q,1\right)},{\bar{t}}_{1}^{\left(q,2\right)},{\bar{t}}_{2}^{\left(q,2\right)},{\bar{t}}_{1}^{\left(q,3\right)},{\bar{t}}_{2}^{\left(q,3\right)},{\bar{t}}_{1}^{\left(q,4\right)},{\bar{t}}_{2}^{\left(q,4\right)}\right]}^{T}$ is the local surface-averaged traction vector, and $K^{\left(q\right)}$ is the local stiffness matrix for a generic subvolume q . However, $K^{\left(q\right)}$ is a non-symmetric matrix, which increases the computational cost of topology optimization problems based on the finite-volume theory when compared to the same approaches based on the finite-element method. Additionally, the surface-averaged tractions are not energetically conjugated with the surface-averaged displacements along the subvolume faces, which leads the $K^{\left(q\right)}$ matrix to be more a pseudo stiffness matrix. Following Araujo et al. (2021), it can be defined a modified local system of equations in terms of resultant forces acting in the edges of a subvolume q , which are energetically conjugated with the surface-averaged displacements, as follows

$$R^{\left(q\right)}={\bar{L}}^{\left(q\right)}{\bar{t}}^{\left(q\right)}={\bar{L}}^{\left(q\right)}K^{\left(q\right)}{\bar{u}}^{\left(q\right)}={\bar{K}}^{\left(q\right)}{\bar{u}}^{\left(q\right)},$$ (5)

where ${\bar{K}}^{\left(q\right)}={\bar{L}}^{\left(q\right)}K^{\left(q\right)}$ is the modified local stiffness matrix, which is found to be a symmetric 8 by 8 matrix, $R^{\left(q\right)}={\left[R_{1}^{\left(q,1\right)},R_{2}^{\left(q,1\right)},R_{1}^{\left(q,2\right)},R_{2}^{\left(q,2\right)},R_{1}^{\left(q,3\right)},R_{2}^{\left(q,3\right)},R_{1}^{\left(q,4\right)},R_{2}^{\left(q,4\right)}\right]}^{T}$ is the local resultant force vector, whose components are illustrated in Figure 2(c), and ${\bar{L}}^{\left(q\right)}$ can be defined as

$${\bar{L}}^{\left(q\right)}=\left[\begin{array}{cccc} L^{\left(q,1\right)} & 0 & 0 & 0\\ 0 & L^{\left(q,2\right)} & 0 & 0\\ 0 & 0 & L^{\left(q,3\right)} & 0\\ 0 & 0 & 0 & L^{\left(q,4\right)} \end{array}\right]\;\text{for}\;L^{\left(q,p\right)}=\left[\begin{array}{cc} L_{p}^{\left(q\right)} & 0\\ 0 & L_{p}^{\left(q\right)} \end{array}\right],$$ (6)

where $L_{1}^{\left(q\right)}=l^{\left(q\right)}$ , $L_{2}^{\left(q\right)}=h^{\left(q\right)}$ , $L_{3}^{\left(q\right)}=l^{\left(q\right)}$ and $L_{4}^{\left(q\right)}=h^{\left(q\right)}$ as illustrated in Figure 1.

Therefore, the modified global system of equations can be written as

$$R=\bar{K}\bar{u},$$ (7)

where $\bar{K}=\sum_{q=1}^{N_{q}}{Q^{\left(q\right)}}^{T}{\bar{K}}^{\left(q\right)}Q^{\left(q\right)}$ is the modified global stiffness matrix, obtained by summing the individual contribution of each subvolume of the discretized domain, with $Q^{\left(q\right)}$ and ${Q^{\left(q\right)}}^{T}$ being the kinematic and static incidence matrices, respectively, R is the global resultant force vector, and $\bar{u}$ is the global surface-averaged displacement vector.

## 3. Topology optimization problems for compliance minimization

A significant portion of the progress in topology optimization has been made through the consideration of compliance minimization problems, whose concepts are well-established in the context of finite-element strategies. In this study, we implement the compliance minimization problem using linear elastic stress analysis based on the finite-volume theory. According to Araujo et al. (2021), the total work done by external loadings and the total strain energy of a deformed structure are equal for quasi-static analysis in the context of the standard finite-volume theory. As a result, the nested topology optimization problem for compliance minimization can be written as

$$\left\{ \begin{array}{c} \text{Find}\;\rho \;\text{which minimizes}\;C\left(\rho \right)=\sum_{q=1}^{N_{q}}{{\bar{u}}^{\left(q\right)}}^{T}{{\bar{K}}^{\left(q\right)}}^{T}{\bar{u}}^{\left(q\right)}=\sum_{q=1}^{N_{q}}E_{q}\left(\rho_{q}\right){{\bar{u}}^{\left(q\right)}}^{T}{{\bar{K}}_{0}^{\left(q\right)}}^{T}{\bar{u}}^{\left(q\right)}\\ \text{subject to}:\\ \frac{V\left(\rho \right)}{\bar{V}}=f\;\\ 0\leq \rho_{q}\leq 1\; \end{array}\right.,$$ (8)

where C(ρ) is the compliance function, defined as twice the work done by external loadings, ρ is the relative density vector, $\rho_{q}$ is the relative density associated with the subvolume q , ${\bar{K}}_{0}^{\left(q\right)}$ is the subvolume modified stiffness matrix for a subvolume with unit Young’s modulus, f is the volume fraction, and V(ρ) and $\bar{V}$ are the material and reference domain volumes, respectively.

**Figure 3.**
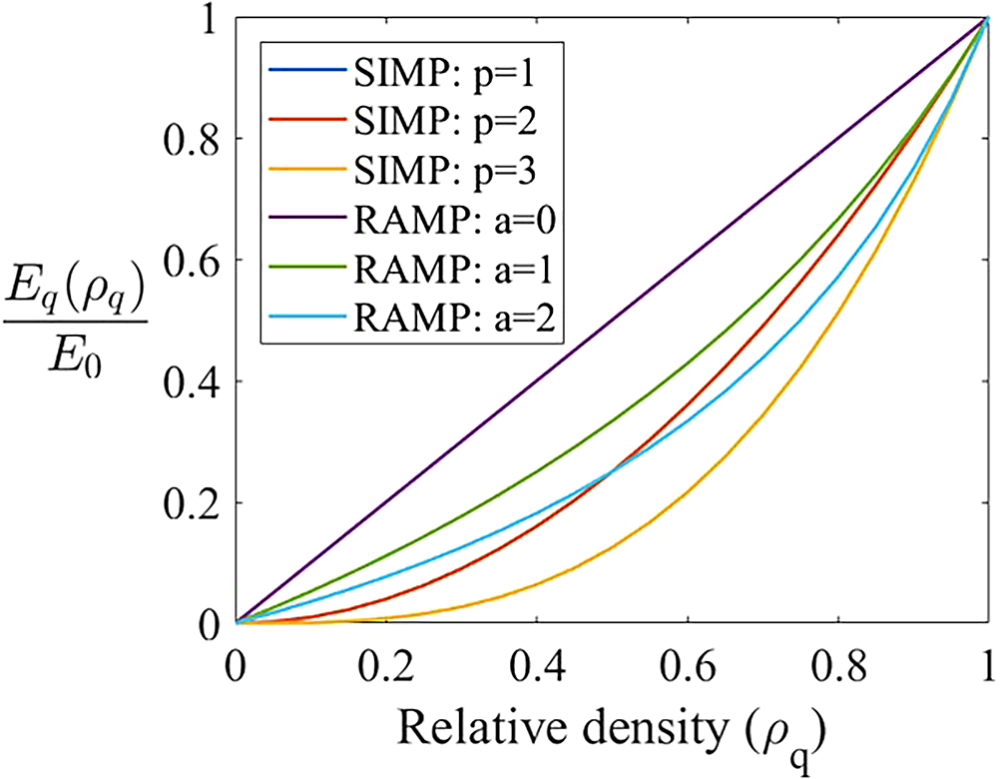
SIMP and RAMP methods’ penalization functions.

The problem presented in Eq. (8) is solved with a nested iterative loop, where at each iteration, the displacement $\bar{u}$ is computed by solving the modified global system of equations presented in Eq. (7). The two major material interpolation functions are implemented in the algorithm: SIMP ( Sigmund, 2007) and RAMP ( Stolpe and Svanberg, 2001). The Young’s modulus $E_{q}\left(\rho_{q}\right)$ of each subvolume can be evaluated by the following expressions:

$$\begin{array}{c} E_{q}\left(\rho_{q}\right)=E_{\min}+\rho_{q}^{p}\left(E_{0}-E_{\min}\right)\;\text{for SIMP}\;\\ E_{q}\left(\rho_{q}\right)=E_{\min}+\frac{\rho_{q}}{1+a\left(1-\rho_{q}\right)}\left(E_{0}-E_{\min}\right)\;\text{for RAMP}\; \end{array},$$ (9)

where p and a are the penalization factors for SIMP and RAMP methods, respectively, $E_{0}$ is the material stiffness, and $E_{\min}$ is the soft (void) material stiffness, which is a non-zero positive low value to avoid the singularity in the stiffness matrix. Figure 3 shows the concavity of the penalization functions performed by the SIMP and RAMP methods as presented by Eq. (9), where the ratio $E_{\min}/E_{0}$ is adopted as $10^{-9}$ . The RAMP method presents a more gradual increase in its concavity when compared to the SIMP method, which softens the numerical response of this method. The function concavity observed in the RAMP method is smoother and presents a slower convergence to the limit relative density values (0 or 1), as observed in the green (RAMP for a=1 ) and blue (RAMP for a=2 ) lines, which incurs in a more gradual convergence for this method. On the other hand, the SIMP method concentrates the relative density values in 0 or 1, as observed in concavity of the orange (SIMP for p=2 ) and yellow (SIMP for p=3 ) lines, promoting a faster convergence to the black and white design.

### 3.1 Objective function gradient

The gradient of the compliance with respect to the subvolume density $\rho_{r}$ can be determined by

$$\frac{\partial C\left(\rho \right)}{\partial \rho_{r}}=\sum_{q=1}^{N_{q}}\left[\frac{\partial {{\bar{u}}^{\left(q\right)}}^{T}}{\partial \rho_{r}}{{\bar{K}}^{\left(q\right)}}^{T}{\bar{u}}^{\left(q\right)}+{{\bar{u}}^{\left(q\right)}}^{T}\frac{\partial {{\bar{K}}^{\left(q\right)}}^{T}}{\partial \rho_{r}}{\bar{u}}^{\left(q\right)}+{{\bar{u}}^{\left(q\right)}}^{T}{{\bar{K}}^{\left(q\right)}}^{T}\frac{\partial {\bar{u}}^{\left(q\right)}}{\partial \rho_{r}}\right].$$ (10)

Employing ${{\bar{K}}^{\left(q\right)}}^{T}={\bar{K}}^{\left(q\right)}$ , the Eq. (10) can be simplified to

$$\frac{\partial C\left(\rho \right)}{\partial \rho_{r}}={{\bar{u}}^{\left(r\right)}}^{T}\frac{\partial {\bar{K}}^{\left(r\right)}}{\partial \rho_{r}}{\bar{u}}^{\left(r\right)}+2\sum_{q=1}^{N_{q}}\left[{{\bar{u}}^{\left(q\right)}}^{T}{\bar{K}}^{\left(q\right)}\frac{\partial {\bar{u}}^{\left(q\right)}}{\partial \rho_{r}}\right].$$ (11)

The Eq. (11) can be rewritten as

$$\frac{\partial C\left(\rho \right)}{\partial \rho_{r}}={{\bar{u}}^{\left(r\right)}}^{T}\frac{\partial {\bar{K}}^{\left(r\right)}}{\partial \rho_{r}}{\bar{u}}^{\left(r\right)}+2{\bar{u}}^{T}\bar{K}\frac{\partial \bar{u}}{\partial \rho_{r}}={{\bar{u}}^{\left(r\right)}}^{T}\frac{\partial {\bar{K}}^{\left(r\right)}}{\partial \rho_{r}}{\bar{u}}^{\left(r\right)}+2R_{p}^{T}\frac{\partial {\bar{u}}_{u}}{\partial \rho_{r}}+2R_{u}^{T}\frac{\partial {\bar{u}}_{p}}{\partial \rho_{r}},$$ (12)

where $R_{p}$ and ${\bar{u}}_{p}$ are the prescribed force and displacement vectors, respectively, and $R_{u}$ and ${\bar{u}}_{u}$ are the unknown force and displacement vectors, respectively. In terms of these vectors, the global system of equations can be decomposed as follows

$$\left[\begin{array}{c} R_{p}\\ R_{u} \end{array}\right]=\left[\begin{array}{cc} {\bar{K}}_{\mathrm{pu}} & {\bar{K}}_{\mathrm{pp}}\\ {\bar{K}}_{\mathrm{uu}} & {\bar{K}}_{\mathrm{up}} \end{array}\right][\begin{array}{c} {\bar{u}}_{u}\;\\ {\bar{u}}_{p} \end{array}].$$ (13)

Once $\partial {\bar{u}}_{p}/\partial \rho_{r}=0$ , the Eq. (12) can be simplified to

$$\frac{\partial C\left(\rho \right)}{\partial \rho_{r}}={{\bar{u}}^{\left(r\right)}}^{T}\frac{\partial {\bar{K}}^{\left(r\right)}}{\partial \rho_{r}}{\bar{u}}^{\left(r\right)}+2R_{p}^{T}\frac{\partial {\bar{u}}_{u}}{\partial \rho_{r}}.$$ (14)

Thus, there are two cases, as described below.

Case 1: prescribed displacement ( $R_{p}=0$ and ${\bar{u}}_{p}\neq 0$ ), which implies in the maximization of C(ρ) .

$$\frac{\partial C\left(\rho \right)}{\partial \rho_{r}}={{\bar{u}}^{\left(r\right)}}^{T}\frac{\partial {\bar{K}}^{\left(r\right)}}{\partial \rho_{r}}{\bar{u}}^{\left(r\right)}$$ (15)

Case 2: prescribed force ( $R_{p}\neq 0$ and ${\bar{u}}_{p}=0$ ), which implies in the minimization of C(ρ) .

$$\frac{\partial C\left(\rho \right)}{\partial \rho_{r}}={{\bar{u}}^{\left(r\right)}}^{T}\frac{\partial {\bar{K}}^{\left(r\right)}}{\partial \rho_{r}}{\bar{u}}^{\left(r\right)}+2{\bar{u}}_{u}^{T}{\bar{K}}_{\mathrm{pu}}\frac{\partial {\bar{u}}_{u}}{\partial \rho_{r}}$$ (16)

Differentiating $R_{p}={\bar{K}}_{\mathrm{pu}}{\bar{u}}_{u}$ in relation to $\rho_{r}$ , follows

$$0=\frac{\partial {\bar{K}}_{\mathrm{pu}}}{\partial \rho_{r}}{\bar{u}}_{u}+{\bar{K}}_{\mathrm{pu}}\frac{\partial {\bar{u}}_{u}}{\partial \rho_{r}}\;∴\;{\bar{K}}_{\mathrm{pu}}\frac{\partial {\bar{u}}_{u}}{\partial \rho_{r}}=-\frac{\partial {\bar{K}}_{\mathrm{pu}}}{\partial \rho_{r}}{\bar{u}}_{u}.$$ (17)

Thus

$$\frac{\partial C\left(\rho \right)}{\partial \rho_{r}}={{\bar{u}}^{\left(r\right)}}^{T}\frac{\partial {\bar{K}}^{\left(r\right)}}{\partial \rho_{r}}{\bar{u}}^{\left(r\right)}-2{\bar{u}}_{u}^{T}\frac{\partial {\bar{K}}_{\mathrm{pu}}}{\partial \rho_{r}}{\bar{u}}_{u}.$$ (18)

Considering ${\bar{u}}_{p}=0$ , follows

$${\bar{u}}_{u}^{T}\frac{\partial {\bar{K}}_{\mathrm{pu}}}{\partial \rho_{r}}{\bar{u}}_{u}={\bar{u}}^{T}\frac{\partial \bar{K}}{\partial \rho_{r}}\bar{u}={{\bar{u}}^{\left(r\right)}}^{T}\frac{\partial {\bar{K}}^{\left(r\right)}}{\partial \rho_{r}}{\bar{u}}^{\left(r\right)}.$$ (19)

This implies

$$\frac{\partial C\left(\rho \right)}{\partial \rho_{r}}={{\bar{u}}^{\left(r\right)}}^{T}\frac{\partial {\bar{K}}^{\left(r\right)}}{\partial \rho_{r}}{\bar{u}}^{\left(r\right)}-2{{\bar{u}}^{\left(r\right)}}^{T}\frac{\partial {\bar{K}}^{\left(r\right)}}{\partial \rho_{r}}{\bar{u}}^{\left(r\right)}=-{{\bar{u}}^{\left(r\right)}}^{T}\frac{\partial {\bar{K}}^{\left(r\right)}}{\partial \rho_{r}}{\bar{u}}^{\left(r\right)},$$ (20)

which results in

$$\frac{\partial C\left(\rho \right)}{\partial \rho_{r}}=-\frac{dE_{r}\left(\rho_{r}\right)}{d\rho_{r}}{{\bar{u}}^{\left(r\right)}}^{T}{\bar{K}}_{0}^{\left(r\right)}{\bar{u}}^{\left(r\right)},$$ (21)

where

$$\begin{array}{c} \frac{dE_{r}\left(\rho_{r}\right)}{d\rho_{r}}=p\rho_{r}^{p-1}\left(E_{0}-E_{\min}\right)\;\text{for SIMP}\;\\ \frac{dE_{r}\left(\rho_{r}\right)}{d\rho_{r}}=\frac{1+a}{{\left[1+a\left(1-\rho_{r}\right)\right]}^{2}}\left(E_{0}-E_{\min}\right)\;\text{for RAMP}. \end{array}$$ (22)

### 3.2 Optimality criteria method

The proposed optimization problem is solved employing the OC method. Following Sigmund (2001) and Andreassen et al. (2011), a heuristic updating scheme identical to the scheme proposed in Bendsøe (1995) can be employed as

$$\rho_{q}^{\mathrm{new}}=\left\{ \begin{array}{ll} \max\left(0,\rho_{q}-m\right), & \text{if}\;\rho_{q}B_{q}^{\eta }\leq \max\left(0,\rho_{q}-m\right),\\ \min\left(1,\rho_{q}+m\right), & \text{if}\;\rho_{q}B_{q}^{\eta }\geq \min\left(1,\rho_{q}+m\right),\\ \rho_{q}B_{q}^{\eta }, & \text{otherwise}\;\\ \; \end{array}\right.,$$ (23)

where m is a positive move-limit, η is a numerical damping factor, and $B_{q}$ is the optimality condition defined as

$$B_{q}=\frac{-\frac{\partial C}{\partial \rho_{q}}}{\lambda \frac{\partial V}{\partial \rho_{q}}},$$ (24)

where the Lagrange multiplier λ can be found by means of a bisection algorithm.

The damping factor can be employed to regularize possible oscillations during the optimization, mainly when no filtering techniques are employed. The parameter η is directly related to the method performance, once this affects the speed variation of $B_{q}^{\eta }$ ( Montes, 2016). A high value for η can accelerate the optimization convergence process, which may cause oscillations in the displacement field for the low-density regions ( Ma et al., 1993). Also, the adoption of minor values of η can prevent divergence in the topology optimization algorithm; however, this results in small changes in the design variables, which leads to a slower convergence process ( Ma et al., 1993). The value of η that provides the faster convergence for the overall process is 1/2, so it is recommended to maintain the damping factor as close as possible of this value.

### 3.3 Mesh-independent filters

As discussed by Araujo et al. (2020b), the topology optimization problem based on the finite-volume theory is a checkerboard-free approach; however, it is observed the occurrence of the mesh-dependency numerical issue. As a result, for topology problems employing the finite-volume theory, filtering techniques are employed to circumvent the mesh dependence issue. Filtering techniques intend to regularize topology optimization numerical issues by using density or sensitivity-based methods. For the density-based methods, each subvolume is redefined by a weighted average of the densities in the subvolume neighborhood, which modifies the sensitivities after the finite-volume analysis. For the strategy based on sensitivity methods, the finite-volume theory analysis is performed, and the sensitivities are consistently calculated; subsequently, they are heuristically recalculated by weighted averaged functions of the sensitivities in the neighboring subvolumes ( Sigmund, 2007).

For the sensitivity-based strategy, the employed filtering technique modifies the subvolumes’ sensitivities as follows

$$\frac{\partial C}{\partial \rho_{q}}=\frac{1}{\max\left(\gamma ,\rho_{q}\right)\sum_{\mathrm{i\epsilon N}}{\hat{H}}_{\mathrm{qi}}}\sum_{\mathrm{i\epsilon N}}{\hat{H}}_{\mathrm{qi}}\rho_{i}\frac{\partial C}{\partial \rho_{i}},$$ (25)

where $\gamma =10^{-3}$ is a small positive real value introduced to avoid division by zero, N is the set of subvolumes i for which the center-to-center distance Δ(q,i) to subvolume q is smaller than the filter radius $r_{\min}$ , and ${\hat{H}}_{\mathrm{qi}}$ is a weight factor evaluated as ( Andreassen et al., 2011)

$${\hat{H}}_{\mathrm{qi}}=\max\left(0,r_{\min}-\Delta \left(q,i\right)\right),$$ (26)

The density filter modifies, besides the sensitivities, the original densities $\rho_{q}$ as follows

$${\hat{\rho }}_{q}=\frac{1}{\sum_{\mathrm{i\epsilon N}}{\hat{H}}_{\mathrm{qi}}}\sum_{\mathrm{i\epsilon N}}{\hat{H}}_{\mathrm{qi}}\rho_{i},$$ (27)

where ${\hat{\rho }}_{q}$ are referred to as the physical densities, as the application of a density filter causes the original densities $\rho_{q}$ to lose their physical meaning ( Sigmund, 2007). When the density filter is employed, the objective function sensitivities with respect to the physical densities ${\hat{\rho }}_{q}$ are given by Eq. (21) once the design variables $\rho_{q}$ are replaced by ${\hat{\rho }}_{q}$ .

## 4. Software description

Top2DFVT is an algorithm developed to obtain optimized topologies using the finite-volume theory for linear elastic continuum structures. The first use of this algorithm performed by Araujo et al. (2020a) was based on the implementation suggested by the top99 code ( Sigmund, 2001), where some operations, such as the filtering procedure and matrices assembly, dramatically increase the computational cost. Therefore, the main features of the top88 code are now explored in this version, such as loop vectorization and memory preallocation, which are strengths of Matlab explored in this program. Additionally, some parts of the code are moved out of the optimization loop, guaranteeing they are only performed once. From the top99neo code, the **fsparse** function is implemented for finite-volume theory matrices assembly, which guarantees a gain of computational efficiency by accelerating the preallocation of these large matrices. The program also explores two new advances in the OC method promoted by the top99neo code. The first advancement incorporates a better approximation for the initial guess of the interval of the Lagrange multiplier λ in the bisection method. This improvement reduces the number of iterations operated by the OC method by suggesting initial values closer to the final solution in the iterative process of the bisection method. The second advancement involves avoiding the application of a filter at each bisection step when checking the volume constraint with the physical field. This alternative reduces the processing time of each bisection iteration and represents another improvement inspired by the top99neo code.

The proposed algorithm is a collection of Matlab functions written in 175 lines, disregarding the commented lines, that implement the design domain, material properties, finite-volume theory analysis, topology optimization, mesh-independency filters, and post-processing. In the data initialization step, the design domain and material properties are defined as inputs to the topology optimization problem, and homogeneous rectangular subvolumes are adopted in the discretized domain. The relative density of each subvolume in the discretized domain is taken as constant. The finite-volume theory analysis is performed for structured meshes considering linear elastic materials for plane stress state. The gradient-based topology optimization problem for compliance minimization is solved employing the OC method, considering a move limit of 0.2. The stopping criterium is set up as follows: 1% of tolerance for the maximum change in the design variables between successive steps. Two mesh-independent filters are implemented: a sensitivity filter and a density filter based on the filtering approaches presented by Andreassen et al. (2011). Finally, the algorithm prints the obtained optimized topology and the investigated numerical aspects, such as the number of iterations, processing time, compliance estimations, etcetera.

### 4.1 Software architecture

The algorithm is initialized by entering the following line in the Matlab command prompt:
Top2DFVT(L,H,nx,ny,volfrac,penal,frad,ft,varargin)


where L and H indicate the horizontal and vertical analysis domain length, respectively, nx and ny are the number of subvolumes in the horizontal and vertical directions, respectively, volfrac is the prescribed volume fraction constraint, penal is the penalty factor, frad is the filter radius, ft specifies whether sensitivity filter (ft = 1), or density filter (ft = 2), or no filter (ft = 0), and varargin activates the use of the *fsparse* routine when set up as ‘fast’. In Top2DFVT.m file, the major sections are default parameters’ declaration, initialization of design variables, domain initialization, local stiffness matrix calculation, material interpolation, filtering initialization, topology optimization iterative process, and post-processing.

**Figure 4.**
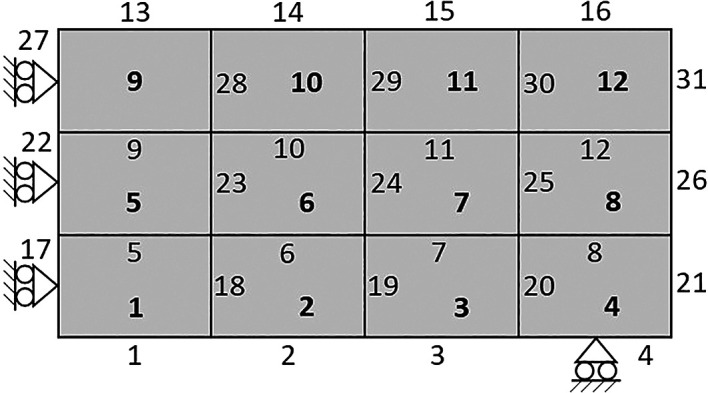
Analysis domain with 12 subvolumes and face indexing.

The default parameters indicate the value of the applied concentrated load, the material Young’s modulus, the soft material stiffness, the Poisson ratio, the type of penalization method, the damping factor, and the maximum number of iterations. Fundamentally, the soft material stiffness must be a minimal value larger than zero, and the type of penalization method can be chosen between ‘SIMP’ or ‘RAMP’ for the material interpolation scheme. While the initialization of the design variables step establishes the discretization of the analysis domain by indexing each subvolume, allocating the relative density, and the volume-constrained gradient matrix. Therefore, the design domain is assumed to be rectangular and discretized in rectangular subvolumes. An example of a coarse mesh composed of 12 subvolumes with four edges per subvolume and two degrees of freedom (DOFs) per face is shown in Figure 4.

The subvolume is indexed row-wise from left to right and down to up, as represented by the bold number shown in Figure 4. Similarly, the subvolume faces are numbered from left to right and down to up, however, the horizontal faces are first indexed, followed by the indexing of the vertical faces, as illustrated in Figure 4. As a result, two DOFs are defined in each subvolume face, where the DOFs 2j−1 and 2j correspond to the horizontal and vertical displacement of face j, respectively. The DOFs assemblage is operated by the subroutine:
[dof,ndof,ijK] = DOFassembly(nx,ny)


where dof is the matrix containing the subvolume DOFs, ndof is the total number of DOFs, and ijK is the indexing matrix employed for the global stiffness matrix assemblage.

The row iK and column jK index vectors are generated by a Kronecker matrix product with a unit vector of 8 lines. The resulting vectors iK and jK are structured so that the iK(i) and jK(j) indices correspond to the assemblage of the stiffness matrix for the subvolume q. The assembly of the global system of equations is performed by employing the *sparse* function in Matlab, which takes three vectors as input arguments: the first and second contain the row and column indices of the non-zero entries, while the third vector contains the entry values of the sparse vectors and matrices. It can be also suggested the use of the *fsparse* routine, developed by Engblom and Lukarski (2016), which enhances the sparse assembly by providing a better ordering of the performed operations. Although Ferrari and Sigmund (2020) have achieved a speedup of 170-250% in the algorithm compared to sparse function on a single-core processor, the performance achieved in our computational environment is similar for both routines. The *fsparse* routine is performed by setting the variable varargin as ‘fast’, while the absence of values for this variable indicates the use of the ‘sparse’ routine.

The structure supporting conditions are prescribed in supp vector by specifying which DOFs of the discretized domain are fixed, while the natural boundary conditions are specified directly in the global force vector F by addressing the DOFs with prescribed loads and their respective magnitude force values. The assemblage of the global stiffness matrix is operated by the function
K = StiffnessAssemblage(sK)


for sK = K0(:)*E(:)’, where K0 is the local stiffness matrix for a unitary elastic modulus obtained with the function
K0 = LocalStiffMatrix(nu,l,h)


and E is the chosen material interpolation scheme. While the local stiffness matrix is symmetric, rounding errors during the assembly of the global stiffness matrix using the *sparse* or *fsparse* commands can cause asymmetry. To correct this, symmetry is enforced at the global level, improving the efficiency of the Matlab backslash (\) command, as recommended by Andreassen et al. (2011).

After solving the global system of equations, the subvolume compliance and its sensitivities are calculated. The objective function value is obtained by adding the individual contribution of each subvolume in the discretized domain, while the subvolume sensitivities are modified considering the aspects of the chosen filtering technique. Subsequently, the design variables are updated by the OC method. The convergence criterium is adopted as 1% of tolerance for the maximum change in design variables. As post-processing step, the investigated numerical aspects are printed, followed by the plotting of the optimized topology. Finally, the processing time is computed for the performed analysis.

## 5. Illustrative examples

**Figure 5.**
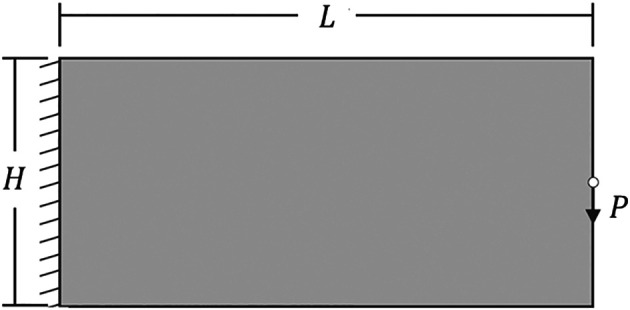
Cantilever deep beam.

The performed example is a cantilever deep beam subject to a concentrated load, as shown in Figure 5. In this case, the vertical and horizontal averaged displacements at the edges of the left border of the structure are fixed, so the supp vector is set up as
supp = unique (dof(1:nx:end-nx+1,7:8))


and the concentrated load is positioned in the middle of the right border in the structure, therefore, the global force vector F is given by
F = sparse (dof (nx*(ny+1)/2,4)’,1,P,ndof,1)


The data initialization is set up as P=−1 , for the applied concentrated load, $E_{0}=1$ , for the Young’s modulus, $E_{\min}=10^{-9}$ , for the soft material stiffness, ν=0.3 , for the Poisson’s ratio, η=1/2 , for the damping factor, move=0.2 , for the move-limit, and maxit=100 , for the maximum number of iterations. For the approaches using the SIMP model, the damping factor is adjusted to 1/2.6 to avoid the oscillatory phenomenon, as discussed by Araujo et al. (2020a, b). The computational environment in terms of programming language and machine can be defined as Matlab R2023a (64-bits) for Windows 11, accompanied by the Optimization and Parallel Computing toolboxes, and processor of 12th Gen Intel(R) Core (TM) i7-1260P 2.10 GHz, RAM 16.0 GB DDR5.

**Figure 6.**
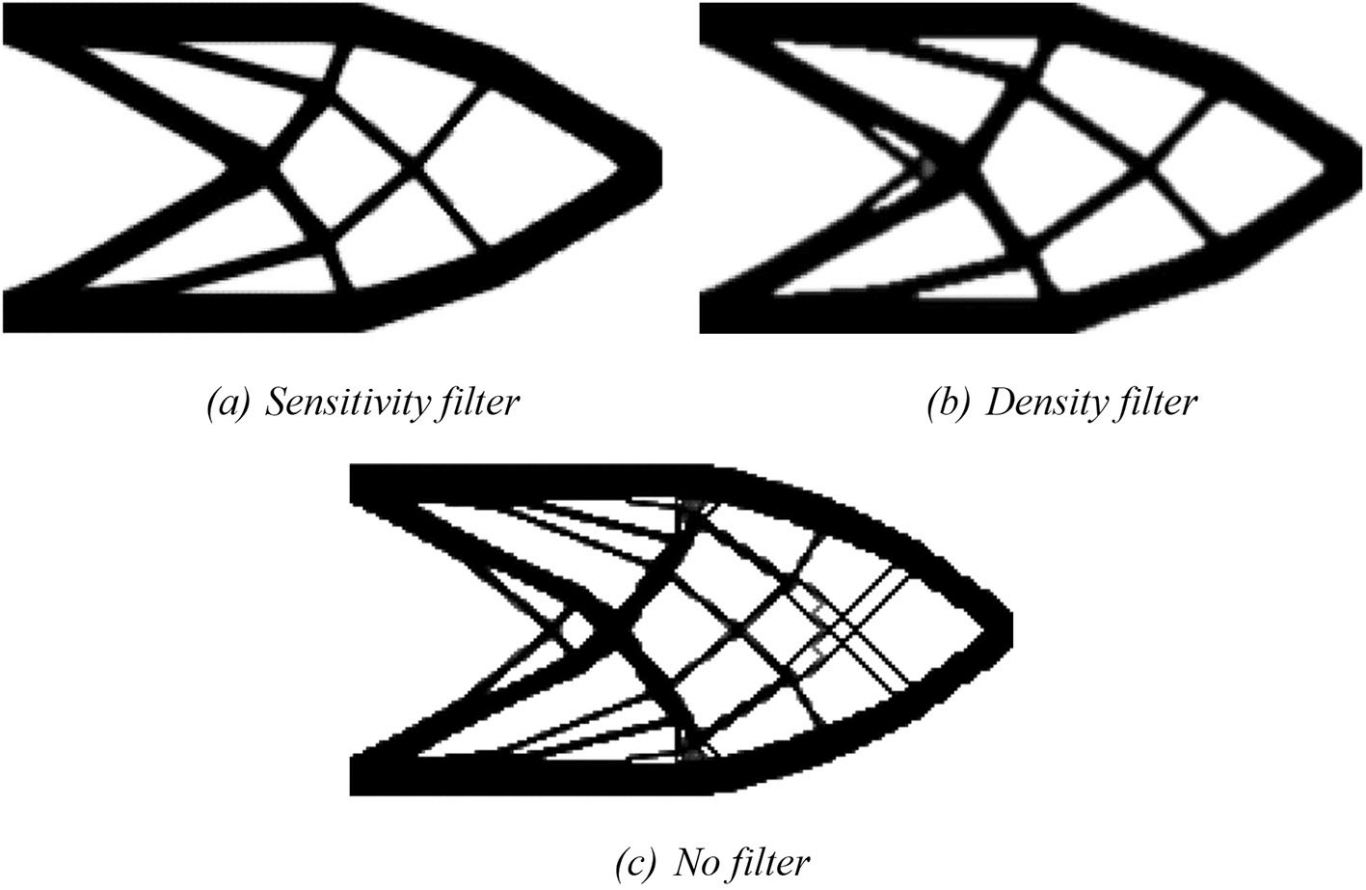
Optimized topologies for the cantilever deep beam employing the SIMP material interpolation.

Considering the same parameters employed by Araujo et al. (2020a) in the filtering scenario, the algorithm can be started by the following command:
Top2DFVT(100,50,202,101,0.4,1:0.5:4,0.71,1)


which consists in the application of the sensitivity filter considering the adjacent subvolumes with a filter radius of 0.71, given by approximately $1.01\sqrt{l_{q}^{2}+h_{q}^{2}}$ , where $l_{q}$ and $h_{q}$ represent the subvolume dimensions, and a volume fraction of 40% of the total volume. The *fsparse* routine can be performed by including varargin = ‘fast’ in the Top2DFVT command. The obtained optimized topologies for the SIMP model are shown in Figure 6, where Figure 6a, 6b, and 6c show the optimized topologies obtained by employing the sensitivity, density, and no filtering techniques, respectively. The investigated numerical aspects are presented in Table 1. In general, the obtained optimized topologies have shown to be checkerboard-free and the employed filtering techniques have qualitatively reduced the mesh dependency issue. Araujo et al. (2020a, b) have already verified these features; however, the current algorithm has obtained similar results by reducing the computational cost by 99.8%. For instance, the same analysis performed for a cantilever deep beam using the sensitivity filter with a mesh of 20,402 subvolumes took 10 hours, 28 minutes, and 37 seconds in Araujo et al. (2020a), while the same analysis employing the Top2DFVT algorithm took only 1 minute and 6 seconds, as shown in Table 1.

**Table 1.** Investigated numerical aspects of the cantilever deep beam with a discretization of 20,402 subvolumes.

SIMP method			
Analysis	Sensitivity filter	Density filter	No filter
Compliance (J)	88.12	91.02	87.90
Filter radius	0.71	0.71	0
Number of iterations	368	577	391
Processing time ( *sparse*)	1min 6s	1min 58s	1min 3s
Processing time ( *fsparse*)	1min 5s	1min 38s	1min 10s
RAMP method			
Analysis	Sensitivity filter	Density filter	No filter
Compliance (J)	85.86	87.69	84.49
Filter radius	0.71	0.71	0
Number of iterations	397	614	451
Processing time ( *sparse*)	1min 1s	1min 38s	1min 16s
Processing time ( *fsparse*)	1min 3s	1min 41s	1min 13s

**Figure 7.**
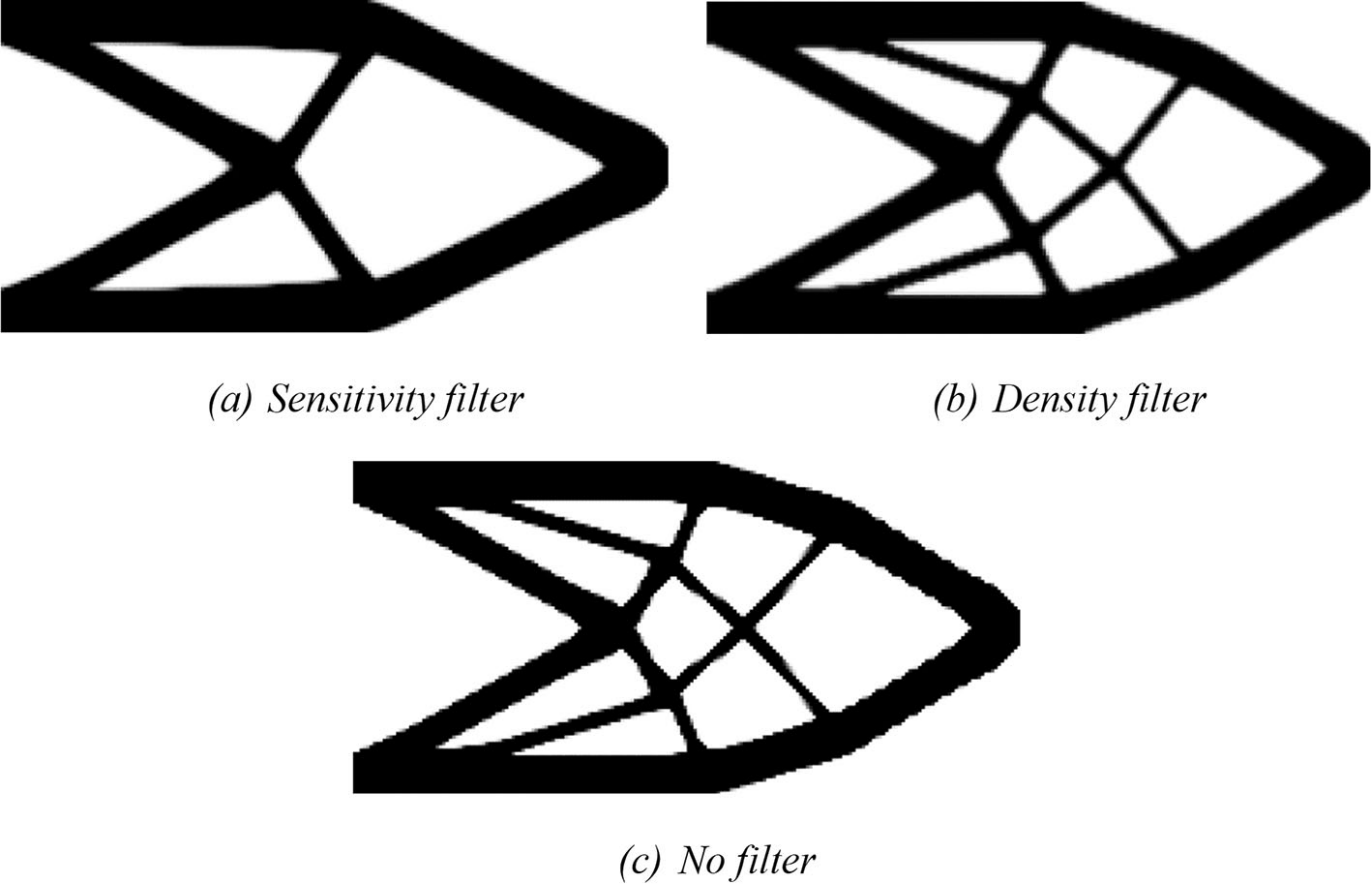
Optimized topologies for the cantilever deep beam employing the RAMP material interpolation.

For the RAMP approach, the penalty factor variable is adjusted to penal = 0:0.5:3, and the variable model is modified to ‘RAMP’. The optimized topologies obtained for the RAMP model are shown in Figure 7, considering the application of the sensitivity filter, Figure 7a, density filter, Figure 7b, and no filtering, Figure 7c. In general, they are checkerboard-free optimized topologies with a reduction in the obtained structural compliance values compared to the optimized topologies generated by the SIMP model, as presented in Table 1. The no-filter approach generated an optimized structure like the optimized topologies obtained by employing the SIMP model and mesh-independent filters. Thus, the RAMP model coupled with the finite-volume theory has shown to be checkerboard-free and mesh-independent for the cantilever deep beam example, which are desired features for manufacturing purposes. In addition, the sensitivity filter for RAMP model has obtained better results by reducing the optimized structural perimeter even more. Table 1 also presents the investigated numerical aspects for the cantilever deep beam example considering the RAMP model. The approach based on the sensitivity filter has presented the lowest number of iterations and computational cost, while the density filter has shown the highest processing time. The minimum value for structural compliance is observed when the no-filtering technique is employed.

For computational efficiency, the ***fsparse*** routine is also implemented; however, for the performed analyses, such a difference in computational cost does not justify using the ***fsparse*** routine. However, a gain in computational performance is observed by around 30% when meshes with size between $10^{5}$ and $10^{6}$ subvolumes are employed. From Table 1, the non-filtering approach has obtained the optimized topologies with the minimum compliance. In contrast, the density filter approach has obtained the optimized topologies with the maximum values for compliance. In general, Top2DFVT provides a platform to perform 2D topology optimization of structures in Matlab, starting from a domain initialization for structured meshes to data post-processing. Several computational tools have been proposed for topology optimization employing analysis domains discretized with essential features for finite-element approaches. As previously discussed, the finite-volume theory is an alternative technique to the finite-element method in the context of topology optimization algorithms. In addition, this is the first contribution to offer an algorithm that shows the implementation of standard finite-volume theory for structured meshes problems in Matlab. This investigation employs the finite-volume theory in topology optimization for compliance minimization problems.

Top2DFVT offers some advantages, such as:

a) It generates checkerboard-free optimized topologies even when a non-filtering approach is employed.
b) It can be applied to medium and large-scale problems, as the implementation and computational performance are suited to these approaches.
c) It employs different material interpolation methods for topology optimization, such as RAMP and SIMP models. When the non-filtering technique is employed, the optimized topologies generated by the RAMP model usually reduce the perimeter compared to those optimized topologies obtained by the SIMP approach.

The Top2DFVT algorithm is currently being employed for educational and research purposes to promote the advantages of the finite-volume theory in the numerical analysis of structures.

## 6. Numerical results

In this contribution, three examples are analyzed employing the compliance minimization problem based on the finite-volume theory for linear elastic materials under plane stress state, where the RAMP and SIMP approaches are employed to interpolate the material stiffness. The investigated examples are a cantilever beam subjected to a concentrated load, a Messerschmitt-Bölkow-Blom (MBB) beam, and an L-bracket beam subject to a concentrated load. Some numerical aspects are also investigated during the analyses, such as the number of iterations, processing time, and compliance estimation. The continued penalization scheme is adopted for the compliance minimization problem, where the penalty factor increases gradually ( ∆p=0.25 ) from 1 to 4 for SIMP and from 0 to 3 for RAMP. A maximum of 200 iterations is assumed for each performed penalty factor along the optimization process.

### 6.1 Cantilever deep beam

**Figure 8.**
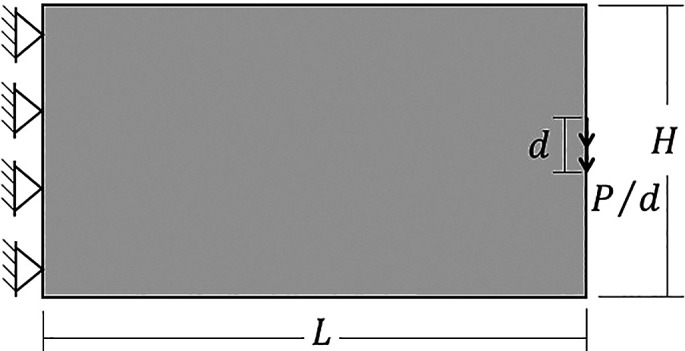
Cantilever deep beam with dimensions and boundary conditions.

A classical problem in topology optimization is the cantilever deep beam, whose analysis domain and boundary conditions are illustrated in Figure 8. In this example, it is observed a region of stress concentration where the concentrated load is applied. The adopted geometrical and physical parameters can be described as H=450 mm, L=900 mm, d=10 mm, P=1000 N, E=200 GPa (Young Modulus), and ν=0.3 (Poisson’s ratio). The proposed optimization problem consists of minimizing the structural compliance, with a volume constraint of 40% of the total volume.

**Figure 9.**
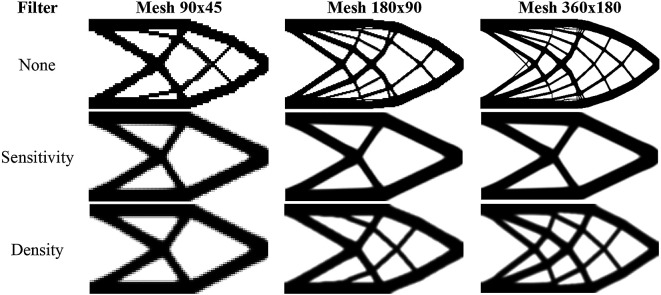
Optimized topologies for the cantilever deep beam obtained by the SIMP approach.

Figure 9 shows the obtained optimized topologies for the approach based on the finite-volume theory considering the SIMP material interpolation method, while Table 2 presents the investigated numerical parameters for each performed analysis. Although the non-filtering approach has obtained the lowest value for the objective function, the sensitivity filter results have presented the lowest computational cost and optimized topologies that better controls the length scale issue, by reducing the formation of thin bars. The density filtering results have shown more thin bars in the optimized topologies when compared to the sensitivity filter, and higher values for the compliance function in the overall investigation. For the SIMP method and considering the non-filtering strategies, the damping factor is adjusted to 1/2.6 to avoid divergence during the optimization process.

**Table 2.** Convergence analysis for the cantilever deep beam problem.

SIMP method					
Analysis	Mesh	Number of iterations	Processing Time	Compliance (J)	Filter radius (mm)
No filter	90x45	371	10s	448.87	0
	180x90	813	4min 11s	391.94	0
	360x180	1183	25min 53s	375.57	0
Sensitivity filter	90x45	213	6s	471.97	15
	180x90	323	1min 25s	406.38	15
	360x180	334	4min 18s	402.01	15
Density filter	90x45	525	15s	491.85	15
	180x90	1450	7min 33s	450.89	15
	360x180	2497	29min 35s	472.49	15
RAMP method					
Analysis	Mesh	Number of iterations	Processing Time	Compliance (J)	Filter radius (mm)
No filter	90x45	545	15s	435.65	0
	180x90	900	3min 12s	382.82	0
	360x180	1164	24min 26s	369.74	0
Sensitivity filter	90x45	350	9s	453.54	15
	180x90	408	54s	394.17	15
	360x180	465	5min 4s	391.36	15
Density filter	90x45	1010	30s	464.51	15
	180x90	2040	4min 44s	420.37	15
	360x180	2309	47min 10s	419.01	15

**Figure 10.**
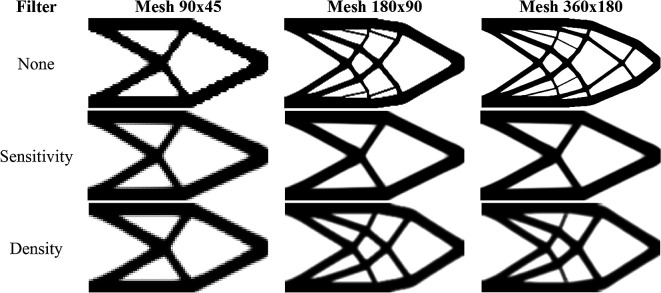
Optimized topologies for the cantilever deep beam obtained by the RAMP approach.

Figure 10 shows the obtained optimized topologies for the approach based on the RAMP method. In general, the RAMP method has obtained checkerboard-free optimized topologies by reducing the structural perimeter when the non-filtering strategy is employed in comparison to the same approach employing the SIMP approach, which is a desired feature for manufacturing purposes. On the other hand, the optimized topologies obtained by the SIMP method usually present a higher structural perimeter by producing more thin bars. Additionally, the RAMP method has obtained a well-defined black-and-white design with lower values for the compliance function, as presented in Table 2.

Table 2 presents the numerical aspects of the performed investigations for the cantilever deep beam example. In general, the RAMP method has presented a higher number of iterations and processing time, although the obtained optimized topologies have presented the lowest values for the objective function. The filter radius is calculated to be slightly higher than $1.01\sqrt{l_{q}^{2}+h_{q}^{2}}$ for the coarse mesh. Therefore, the optimized topology obtained for the finest mesh employing the sensitivity filter is very similar to that obtained for the coarse mesh without filtering techniques. This is only possible because the finite-volume theory is a checkerboard-free numerical technique in topology optimization algorithms.

### 6.2 Half MBB beam

**Figure 11.**
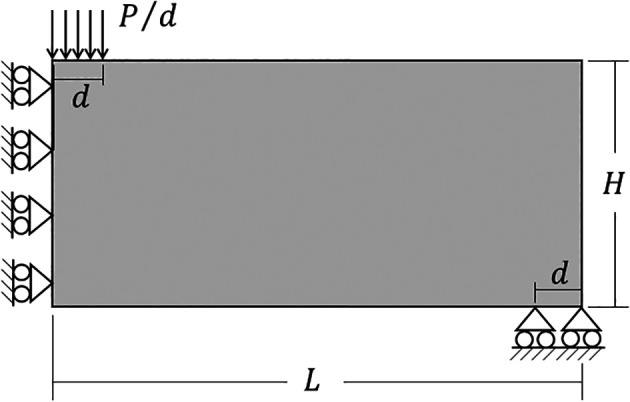
Half-MBB beam with dimensions and boundary conditions.

Other classical problem for topology optimization of continuum structures is the Messerschmitt-Bölkow-Blom (MBB) beam. In this case, only half of the structure is analyzed as shown on Figure 11, where the geometric and physical parameters are taken as H=300 mm, L=900 mm, d=10 mm, P=1000 N, E=78 GPa (Young Modulus), and ν=0.25 (Poisson’s ratio). The volume fraction for the minimum compliance optimization problem is assumed as 40% of the total structure volume.

**Figure 12.**
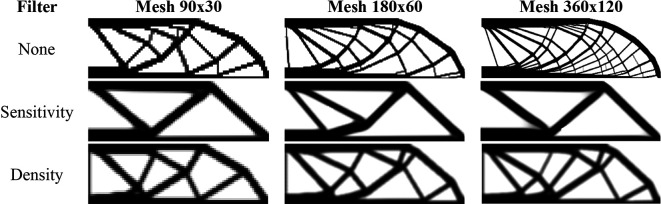
Optimized topologies for the MBB beam obtained by the SIMP approach.

Figure 12 shows the optimized topologies obtained considering the application of the SIMP method, while Table 3 presents the investigated numerical aspects for each performed analysis. The topology optimization technique considers the non-filtering, sensitivity, and density filtering scenarios. The adopted filter radius is slightly higher than half of the subvolume’s diagonal length for the coarsest mesh, which can be written as $1.01\sqrt{l_{q}^{2}+h_{q}^{2}}$ and approximated by 15 mm. The no-filter analysis generally generates topologies with more thin bars, while the sensitivity filter obtains cleaner topologies with a reduced structural perimeter. Besides, the density filter has not presented the same efficiency as the sensitivity filter in reducing the structural perimeter in the final optimized topology, and the obtained compliance is higher when compared to the other approaches. Regarding computational cost, the sensitivity filter approach obtained the lowest processing time and number of iterations, while the density filter approach presented the highest processing time and number of iterations.

**Table 3.** Convergence analysis for the half MBB beam problem.

SIMP method					
Analysis	Mesh	Number of iterations	Processing Time	Compliance (J)	Filter radius (mm)
No filter	90x30	538	10s	3160.01	0
	180x60	804	1min 19s	2873.54	0
	360x120	1352	11min 4s	2759.59	0
Sensitivity filter	90x30	410	8s	3174.88	15
	180x60	533	48s	3050.52	15
	360x120	921	7min 26s	2989.76	15
Density filter	90x30	843	15s	3586.61	15
	180x60	1819	4min 59s	3524.15	15
	360x120	2490	20min 51s	3560.40	15
Analysis	Mesh	Number of iterations	Processing Time	Compliance (J)	Filter radius (mm)
No filter	90x30	811	15s	2921.81	0
	180x60	1315	2min 50s	2731.09	0
	360x120	1617	17min 14s	2654.87	0
Sensitivity filter	90x30	538	10s	3049.43	15
	180x60	747	1min 51s	2935.32	15
	360x180	1040	8min 27s	2923.79	15
Density filter	90x30	1191	21s	3174.77	15
	180x60	2289	4min 11s	3084.24	15
	360x120	2450	21min 16s	3087.59	15

**Figure 13.**
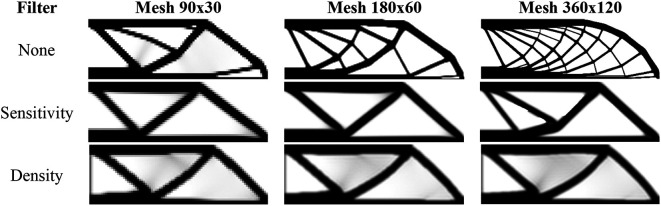
Optimized topologies for the MBB beam obtained by the RAMP approach.

Figure 13 shows the optimized topologies for the analyses employing the RAMP method, where the adopted filter radius is the same as those employing the SIMP method. The RAMP method has generally obtained optimized topologies with better control of the structural perimeter, even when the non-filtering technique is employed. Additionally, the optimized topology obtained for the coarse mesh without filtering techniques is geometrically close to the optimized topologies for the finest mesh employing filtering strategies. Therefore, the results obtained for the coarse mesh in the no-filter strategy employing the RAMP method could be adopted as the solution for the optimization problem. Table 3 presents the investigated numerical aspects, where the number of iterations and processing time are usually higher for this method when compared to the SIMP approach.

### 6.3 L-bracket beam

**Figure 14.**
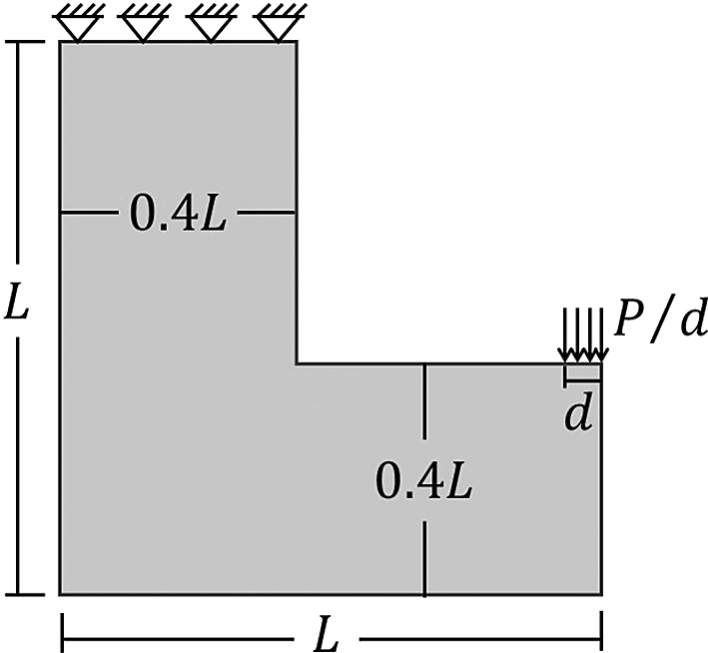
L-bracket beam domain, with d=5 cm, P=200 kN, and L=1 m.

Another analyzed topology optimization problem for stress concentration in two-dimensional structures is the L-bracket beam, whose analysis domain and boundary conditions are illustrated in Figure 14. In the L-bracket beam problem, it is observed a high level of stress concentration in the corner, which is important to check how the new Top2DFVT code leads to these kinds of topology optimization problems. The employed geometric parameters for this beam are assumed as d=5 cm, L=100 cm, and P=200 kN, while the adopted material properties are E=70 GPa (elastic moduli) and ν=0.25 (Poisson’s ratio). The proposed optimization problem consists of minimizing the structural compliance function under a volume constraint of 40% of the total volume.

**Figure 15.**
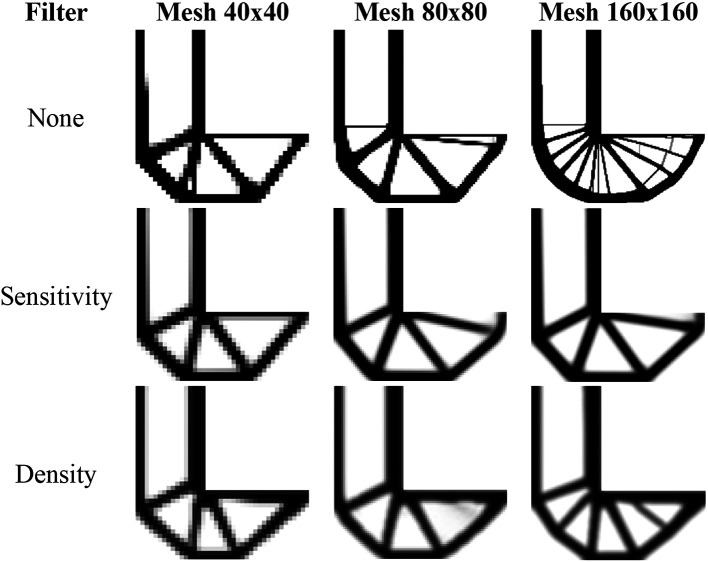
Optimized topologies for the L-bracket beam obtained by the SIMP approach.

Figure 15 shows the optimized topologies obtained by the SIMP approach for the L-bracket beam problem, considering the absence of filtering techniques and the implementation of the sensitivity and density filters, respectively. The sensitivity filter has reduced the formation of thin bars along the optimized topologies, while the density filter has obtained irregular optimized topologies with the appearance of substantial gray regions. On the other hand, the no-filter strategy has generated well-defined optimized topologies with more thin bars, especially when compared to the sensitivity filter strategy. As Araujo et al. (2020a) suggested, the damping factor is adjusted to 1/2.6 for all performed approaches employing the SIMP to guarantee the absence of the oscillatory phenomenon to any employed filter radius. Table 4 presents the investigated numerical aspects for the performed analyses employing the SIMP method.

**Table 4.** Convergence analysis for the L-bracket beam problem.

SIMP method					
Analysis	Mesh	Number of iterations	Processing Time	Compliance (J)	Filter radius (cm)
No filter	40x40	404	4s	4469.64	0
	80x80	698	35s	17388.97	0
	160x160	946	4min 11s	68743.32	0
Sensitivity filter	40x40	274	2s	4794.02	3.6
	80x80	226	10s	19461.58	3.6
	160x160	155	39s	81058.11	3.6
Density filter	40x40	653	6s	6149.43	3.6
	80x80	1140	1min 51s	25552.58	3.6
	160x160	2585	11min 19s	115640.37	3.6
RAMP method					
Analysis	Mesh	Number of iterations	Processing Time	Compliance (J)	Filter radius (cm)
No filter	40x40	272	2s	4434.37	0
	80x80	692	35s	17241.14	0
	160x160	894	6min 38s	67478.81	0
Sensitivity filter	40x40	278	2s	4714.58	3.6
	80x80	202	10s	19325.52	3.6
	160x160	226	51s	77303.15	3.6
Density filter	40x40	590	5s	5848.89	3.6
	80x80	1640	1min 41s	24364.83	3.6
	160x160	2546	20min 6s	98444.69	3.6

**Figure 16.**
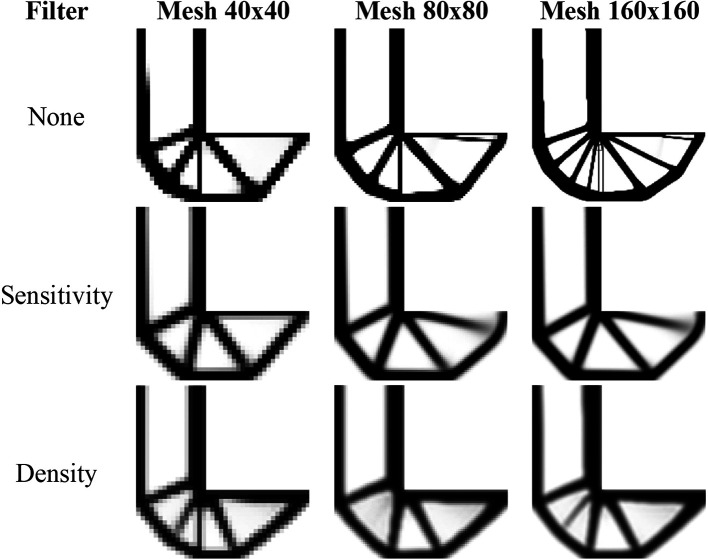
Optimized topologies for the L-bracket beam obtained by the RAMP approach.

Figure 16 presents the obtained optimized topologies by the RAMP method for the L-bracket beam problem. The RAMP method has generally reduced the formation of thin bars, demonstrating less sensitivity with the adopted meshes. As in the SIMP method, the sensitivity filter has shown to be more efficient by reducing the formation of thin bars in the optimized topologies, and the density filter has obtained more irregular topologies with the presence of gray regions. The RAMP method is more stable numerically, and the adopted damping factor is 1/2, which guarantees a faster convergence for the analyses. However, the number of iterations is usually higher for the RAMP method.

Table 4 presents the investigated numerical aspects of the optimized structures, such as the total number of iterations, processing time, and compliance estimation. When the sensitivity filter is employed, there is a remarkable decrease in the number of iterations and computational costs. However, the obtained values for structural compliance are lower when the non-filtering strategy is performed. In general, the RAMP method has been shown to efficiently produce checkerboard-free optimized topologies with lower values for structural compliance. Thus, these results demonstrate the proposed approach’s efficiency and justify its use in topology optimization problems of continuum elastic structures since it better controls numerical issues associated with checkerboard and length scale. The filter radius is slightly higher than $1.01\sqrt{l_{q}^{2}+h_{q}^{2}}$ for the coarse mesh. As a result, the filter guarantees the absence of mesh dependency, especially when the RAMP method or the sensitivity filter are employed.

## 7. Conclusions

This study introduces the Top2DFVT, an innovative Matlab algorithm tailored for the topology optimization of two-dimensional elastic structures via the finite-volume theory. This contribution addresses compliance minimization problems, presenting a checkerboard-free methodology that mitigates numerical instabilities like mesh dependence and local minima, commonly encountered in gradient-based optimization techniques. The algorithm showcases improved computational efficiency and the ability to generate optimized topologies for medium to large-scale problems by employing two material interpolation schemes, SIMP and RAMP, alongside sensitivity and density filters. Such advancements facilitate the design of high-performance structures with potential applications in various engineering domains.

This algorithm can provide checkerboard-free optimized topologies and reduce mesh dependence or length scale issues, mainly when the RAMP method is employed. The optimized topologies obtained without filtering techniques for the coarse meshes and employing the RAMP method are similar to those obtained with filtering strategies for the finer meshes. Usually, filtering techniques are based on image processing that geometrically changes the sensitivity or the relative density values. Therefore, obtaining optimized structures without filtering techniques provides more reliable and efficient designs. Besides, the optimized topologies without filtering strategies are well-defined “black and white” designs, where intermediate values of relative densities are reduced.

The approach based on the finite-volume theory is also performed by employing a sensitivity filter to solve problems related to mesh dependence and length scale issues. The adopted strategy to define the filter radius consists of using approximately the subvolume’s or element’s diagonal of the coarse mesh. The continued penalization scheme is adopted for the compliance minimization problem, which guarantees a gradual convergence for the overall process. When the SIMP method is employed, the OC method’s damping factor can be adjusted to 1/2.6 to avoid divergence during the optimization process, especially when non-filtering strategies are employed.

In conclusion, this study presents a novel approach to topology optimization using the finite-volume theory and significantly contributes to the field by addressing and overcoming inherent numerical challenges. The Top2DFVT algorithm represents a pivotal advancement in optimizing elastic structures, promising more reliable and efficient design solutions. The authors’ efforts in developing and sharing this tool underscore the collaborative spirit of the research community, aiming to broaden the understanding and application of topology optimization in engineering.

This work sets a new benchmark for future research, encouraging further exploration and development of optimization techniques. By providing a robust and efficient tool in Top2DFVT, the authors offer valuable resources for educators, researchers, and practitioners alike, fostering innovation and excellence in engineering design.

### Ethics and consent

Ethical approval and consent were not required.

## Data Availability

No data are associated with this article.

## Software availability

All the presented results can be reproduced using the codes available in the GitHub links ( https://github.com/fvt7782/Top2DFVT, for Matlab implementation, and https://github.com/fvt7782/Top2DFVT_Oct, for GNU Octave implementation) and by following the instructions presented in this article. For the GNU Octave implementation, the fsparse speedup is not available. The alternative version of the code using the GNU Octave platform has a few changes compared to the original Matlab code. The processing times for the GNU Octave code are higher than those for the Matlab code, but all the optimized topologies shown in the article can be generated with the GNU Octave code.

• **Source code available from**: https://github.com/fvt7782/Top2DFVT, for Matlab implementation, and https://github.com/fvt7782/Top2DFVT_Oct, for GNU Octave implementation.
• **Archived source code at time of publication**: https://doi.org/10.5281/zenodo.11391501, for Matlab implementation, and https://doi.org/10.5281/zenodo.12551626, for GNU Octave implementation.
• **Licenses**: The 2-Clause BSD License and CC-BY 4.0.
