# Supplementary material for: TOP2DFVT: An Efficient Matlab Implementation for Topology Optimization based on the Finite-Volume Theory
Source: F1000Res. 2024 Oct 22;13:805. Originally published 2024 Jul 16. [Version 2] doi: 10.12688/f1000research.150945.2 (PMC11512726; doi:10.12688/f1000research.150945.2)
Supplement: Peer review history

# Reviewer report: Reviewer response for version 2

**DOI:** 10.5256/f1000research.173411.r333833

**Published:** 2024-10-30

**Authors:** Volnei Tita

**Affiliations:** 1 University of Sao Paulo, Sao Paulo, Brazil

I do not have further comments to make.

Is the work clearly and accurately presented and does it cite the current literature?

Yes

If applicable, is the statistical analysis and its interpretation appropriate?

Not applicable

Are all the source data underlying the results available to ensure full reproducibility?

Yes

Is the study design appropriate and is the work technically sound?

Yes

Are the conclusions drawn adequately supported by the results?

Yes

Are sufficient details of methods and analysis provided to allow replication by others?

Yes

Reviewer Expertise:

Computational Analysis of Composite Structures

I confirm that I have read this submission and believe that I have an appropriate level of expertise to confirm that it is of an acceptable scientific standard.

# Reviewer report: Reviewer response for version 2

**DOI:** 10.5256/f1000research.173411.r333832

**Published:** 2024-10-29

**Authors:** Minh Ngoc Nguyen

**Affiliations:** 1 Duy Tan Research Institute for Computational Engineering, Duy Tan University, Ho Chi Minh city, Vietnam

The authors have addressed all of my comments.

Is the work clearly and accurately presented and does it cite the current literature?

Yes

If applicable, is the statistical analysis and its interpretation appropriate?

Not applicable

Are all the source data underlying the results available to ensure full reproducibility?

Yes

Is the study design appropriate and is the work technically sound?

Yes

Are the conclusions drawn adequately supported by the results?

Yes

Are sufficient details of methods and analysis provided to allow replication by others?

Yes

Reviewer Expertise:

Topology optimization

I confirm that I have read this submission and believe that I have an appropriate level of expertise to confirm that it is of an acceptable scientific standard.

# Reviewer report: Reviewer response for version 2

**DOI:** 10.5256/f1000research.173411.r333831

**Published:** 2024-10-26

**Authors:** Yingjun Wang

**Affiliations:** 1 South China University of Technology, Guangzhou, Guangdong, China

The authors have addressed all of my comments.

Is the work clearly and accurately presented and does it cite the current literature?

Yes

If applicable, is the statistical analysis and its interpretation appropriate?

Yes

Are all the source data underlying the results available to ensure full reproducibility?

Partly

Is the study design appropriate and is the work technically sound?

Yes

Are the conclusions drawn adequately supported by the results?

Yes

Are sufficient details of methods and analysis provided to allow replication by others?

Yes

Reviewer Expertise:

topology optimization

I confirm that I have read this submission and believe that I have an appropriate level of expertise to confirm that it is of an acceptable scientific standard.

# Reviewer report: Reviewer response for version 1

**DOI:** 10.5256/f1000research.165561.r313235

**Published:** 2024-09-04

**Authors:** Volnei Tita

**Affiliations:** 1 University of Sao Paulo, Sao Paulo, Brazil

The paper consists of providing a MATLAB code based on Finite-Volume Theory (named Top2DFVT), which can perform 2D topology optimization of structures by using SIMP and RAMP algorithms. On one hand, the manuscript brings relevant results, mitigating some numerical instabilities, such as checkerboard and mesh dependence issues. By another hand, there are some important aspects related to the manuscript and to the implemented code, which need to be better clarified by the authors as follows:

 (*) 1. Introduction

 - The authors claim that “…this is the first time a platform for optimizing structures using the finite-volume theory…”. It is very hard to confirm that there is not any work in the World previously published in a journal or conference in this field. Thus, it is recommended that the authors modify the text by the inclusion of “Based on the authors’ knowledge, this is the first time a platform…”.

 - The authors mentioned about computational cost, comparing the previous version of the code approached in Araujo et al. (2020a) with the present one. However, it would be very important to compare and discuss advantages and disadvantages with other codes. For example, usually, FORTRAN codes run faster than MALTAB codes. In fact, in the final of Section 5, the authors presented some advantages, but it is not possible to find drawbacks. 

 (*) 2. Finite-volume theory

 - In the Eq (4), K is a non-symmetric matrix, then the computational cost of topology optimization based on the FVT is higher than based on the FEM. The authors should comment quantitative values, expressing how slow is the process based on the FVT.

 (*) 6. Numerical results

 - The authors analyzed 3 Case Studies (cantilever deep beam; half MBB beam; L-bracket beam) to show the potentialities of the developed MATLAB code. But they are invited to analyze one of the case studies presented in the paper cited below:

 A concurrent fibre orientation and topology optimisation framework for 3D-printed fibre-reinforced composites -

 (Almeida Jr. J, et al., 2023 [Ref 1])

 In other words, they should compare the results obtained by the Top2DFVT with the results discussed in the paper.

Is the work clearly and accurately presented and does it cite the current literature?

Yes

If applicable, is the statistical analysis and its interpretation appropriate?

Not applicable

Are all the source data underlying the results available to ensure full reproducibility?

Yes

Is the study design appropriate and is the work technically sound?

Yes

Are the conclusions drawn adequately supported by the results?

Yes

Are sufficient details of methods and analysis provided to allow replication by others?

Yes

Reviewer Expertise:

Computational Analysis of Composite Structures

I confirm that I have read this submission and believe that I have an appropriate level of expertise to confirm that it is of an acceptable scientific standard, however I have significant reservations, as outlined above.

## References

[1] : A concurrent fibre orientation and topology optimisation framework for 3D-printed fibre-reinforced composites. *Composites Science and Technology* .2023;232: 10.1016/j.compscitech.2022.109872 10.1016/j.compscitech.2022.109872

# Response

**Published:** 2024-10-13

**Authors:** Marcio Cavalcante

The authors have many thanks to the reviewer for their valuable comments. The manuscript has been revised in accordance with the comments, and detailed revisions are listed below point by point.

***Comments to the Authors:***

 The paper consists of providing a MATLAB code based on Finite-Volume Theory (named Top2DFVT), which can perform 2D topology optimization of structures by using SIMP and RAMP algorithms. On one hand, the manuscript brings relevant results, mitigating some numerical instabilities, such as checkerboard and mesh dependence issues. By another hand, there are some important aspects related to the manuscript and to the implemented code, which need to be better clarified by the authors as follows:

 1) Introduction

 - The authors claim that “…this is the first time a platform for optimizing structures using the finite-volume theory…”. It is very hard to confirm that there is not any work in the World previously published in a journal or conference in this field. Thus, it is recommended that the authors modify the text by the inclusion of “Based on the authors’ knowledge, this is the first time a platform…”.

***Response: The authors agreed with the reviewer, and the following modification was made to the manuscript:***

 In addition, **based on the authors’ knowledge,** this is the first time a platform for optimizing structures using the finite-volume theory can be applied to medium and large-scale problems, besides obtaining checkerboard-free and mesh-independent designs.

 1) Introduction

 - The authors mentioned about computational cost, comparing the previous version of the code approached in Araujo et al. (2020a) with the present one. However, it would be very important to compare and discuss advantages and disadvantages with other codes. For example, usually, FORTRAN codes run faster than MALTAB codes. In fact, in the final of Section 5, the authors presented some advantages, but it is not possible to find drawbacks.

***Response: The authors agreed with the reviewer, and the following texts and figures were added to the manuscript with results obtained by the top99neo algorithm, discussing the advantages and disadvantages of the Top2DFVT code:***

 The algorithm known as top99neo, proposed by Ferrari and Sigmund in 2020, is used as a benchmark source for the traditional finite-element method for bilinear elements. The algorithm uses the continued penalization scheme, gradually increasing the penalty factor (∆p=0.25) from 1 to 4, similar to the finite-volume theory approach. Each penalty factor is subjected to 25 iterations. The Heaviside projection parameters are updated throughout the optimization process. The parameter β starts at 2 and increases to 16 by an increment of ∆β=2 every 25 iterations. Figure 12 shows the optimized topologies obtained using the density filter. The filter radius changes to reflect the exact size of the filter radius employed in the finite-volume theory approaches for each mesh size (1.5, 3, and 6, respectively). The optimized topologies obtained using the finite-element approach are more mesh-dependent, even when filtering strategies are employed, as compared to the optimized topologies obtained using the finite-volume theory approach, especially in the case of the results with the sensitivity filter. The computational cost for the finest mesh is 2 minutes and 30 seconds, with a maximum of 350 iterations. The total number of degrees of freedom for the finite-element approach is 132132, while the finite-volume theory approach has a total of 260280 degrees of freedom, which partially explains the difference in computational costs. As the top99neo algorithm employs finite elements with unitary dimensions, it is not feasible to compare the obtained values for the objective function of the optimized topologies.

**See Figure 12.** Optimized topologies for the cantilever deep beam employing the top99neo algorithm.( URL)

 Figure 16 shows the optimized topologies obtained employing the top99neo algorithm, where the penalty factor increases gradually (∆p=0.25) from 1 to 4 after every 25 iterations until a maximum of 350 iterations, and the beta parameter increases gradually from 2 to 16 (∆β=2), after every 25 iterations, similarly to the cantilever deep beam example. The optimized topologies shown in Figure 16 are mesh dependents even when filtering strategies are employed. The filter radius is assumed to be 1.5 for the 90x30 mesh, 3 for the 180x60 mesh, and 6 for the 360x120 mesh. When a similar filter radius is employed in the context of the finite-volume theory, this technique demonstrates a less mesh sensitivity behavior when compared to finite element-based strategies. Regarding computational efficiency, the top99neo code has presented a processing time of 86 seconds for the finest mesh. Generally, the Top2DFVT algorithm presents a higher computational cost when compared to the Q4 finite element-based algorithm. The number of degrees of freedom partially explains this higher computational cost once the total number of degrees of freedom for the finest mesh considering the top99neo algorithm is 87362, while in the Top2DFVT algorithm, the total number of degrees of freedom for the same analysis is 173760. Furthermore, the number of iterations for this example is relatively high for the finite-volume theory approaches once the top99neo algorithm adopts a maximum of 350 iterations.

**See Figure 16.** Optimized topologies for the MBB beam employing the top99neo algorithm.( URL)

 The top99neo algorithm is also performed considering the L-bracket beam problem, and the obtained optimized topologies can be observed in Figure 20. As in the previous examples, the same numerical parameters are employed for the physical model, such as the gradual increase in the penalty factor, from 1 to 4 with ∆p=0.25, and in the Heaviside Projection β parameter, from 2 to 16 with ∆β=2. In terms of computational cost, the top99neo code has presented a processing time of 1 minute and 1 second for the finest mesh, considering a total of 350 iterations, while the Top2DFVT algorithm has presented a processing time of 39 seconds with a total of 155 iterations for of the SIMP method and the sensitivity filter. This difference in processing time can be partially explained by the difference in the total number of iterations observed during the topology optimization analyses. For this example, the Top2DFVT algorithm, considering the sensitivity filter approach, presents a reduced number of iterations for convergence.

**See Figure 20.** Optimized topologies for the L-bracket beam employing the top99neo algorithm.( URL)

 2. Finite-volume theory

 - In the Eq (4), K is a non-symmetric matrix, then the computational cost of topology optimization based on the FVT is higher than based on the FEM. The authors should comment quantitative values, expressing how slow is the process based on the FVT.

***Response: The local stiffness matrix in Eq. (4) is non-symmetric, which can lead to higher computational costs compared to FEM-based approaches. To address this, we adopted a modified symmetric stiffness matrix, as shown in Eq. (5), motivated by physical considerations. Specifically, the modified local stiffness matrix allows the adoption of energetically conjugated quantities, i.e., the surface-averaged displacements and the resultant forces acting on the subvolume faces. To clarify this, the following text was added to the manuscript:***

 The modified global stiffness matrix allows the adoption of energetically conjugated quantities, i.e., the surface-averaged displacements and the resultant forces acting on the subvolume faces. This adjustment not only guarantees better physical consistency but also improves computational efficiency by enabling the use of solvers optimized for symmetric systems. As a result, the time required to solve the modified global system of equations is significantly reduced, bringing the computational cost closer to the finite element method-based approaches while retaining the benefits of the finite-volume theory in terms of numerical stability and checkerboard-free solutions.

 6. Numerical results

 - The authors analyzed 3 Case Studies (cantilever deep beam; half MBB beam; L-bracket beam) to show the potentialities of the developed MATLAB code. But they are invited to analyze one of the case studies presented in the paper cited below:

 A concurrent fibre orientation and topology optimisation framework for 3D-printed fibre-reinforced composites - (Almeida Jr. J, et al., 2023 [Ref 1])

 In other words, they should compare the results obtained by the Top2DFVT with the results discussed in the paper.

***Response: The authors appreciate the reviewer's suggestion to include an additional case study based on the work of Almeida et al. (2023). While their paper presents noteworthy results, particularly in the concurrent optimization of fibre orientation and topology for 3D-printed composites, the authors have opted not to expand the scope of the current manuscript with further case studies. The focus of this article is to present the capabilities of the Top2DFVT code within the context of traditional isotropic materials, and the authors believe the selected case studies effectively demonstrate the tool's potential.***

***However, the authors recognize the value of comparing results with those presented in Almeida et al. (2023) and intend to explore these comparisons in future publications. For now, the authors have chosen to include the following text in the introduction of the manuscript, highlighting recent advancements in topology optimization for anisotropic composites:***

 Recent advances in topology optimization have expanded to include anisotropic composites, optimizing both material distribution and fibre orientation. Almeida et al. (2023) introduced a framework for concurrent optimization of topology and fibre orientation in 3D-printed fibre-reinforced composites, effectively minimizing compliance. This approach, particularly for materials like onyx, enhances stiffness and strength, offering significant benefits for additive manufacturing of high-performance and lightweight structures.

***Added reference:***

 Almeida JHS Jr, Christoff BG, Tita V, St-Pierre L. A concurrent fibre orientation and topology optimisation framework for 3D-printed fibre-reinforced composites. Composites Science and Technology. 2023;232:109872. doi: 10.1016/j.compscitech.2022.109872.

# Reviewer report: Reviewer response for version 1

**DOI:** 10.5256/f1000research.165561.r313232

**Published:** 2024-08-26

**Authors:** Minh Ngoc Nguyen

**Affiliations:** 1 Duy Tan Research Institute for Computational Engineering, Duy Tan University, Ho Chi Minh city, Vietnam

Comments for

**Top2DFVT** **: An efficient Matlab Implementation for topology optimization based on Finite-Volume theory**

 The manuscript presents a Matlab implementation for topology optimization based on Finite Volume theory for 2D compliance problems.

 In general, the manuscript is interesting and is very useful for beginners of topology optimization. The Matlab code is fast, due to vectorization and memory pre-allocation, in a manner similar to the code top88 (Andreassen E, et al., 2011 [Ref 1]). Compared to the previous works by the same group of authors, computational time is reduced significantly. However, there are many unclear information that needs thorough revision by the authors

 1. Although the approach may not require filter to avoid checkerboard pattern, it is clearly observed in the results that when no filter is applied, many thin components appear in the optimized design. Such thin components may be suffered from high stress values. In fact, as in the literature, filter also contributes to length-scale control. The authors should discuss this aspect.

 2. When there is no filter, it seems that clear “black and white” results could be obtained. Nevertheless, the authors should compute the non-discreteness values to quantitatively demonstrate this aspect.

 On the other hand, when filter (either sensitivity filter or density filter) is applied, gray elements appear. In order to reduce the gray elements and increase the discreteness of the solution, volume-preserving Heaviside projection is commonly employed. The authors should also mention this.

Is the work clearly and accurately presented and does it cite the current literature?

Yes

If applicable, is the statistical analysis and its interpretation appropriate?

Not applicable

Are all the source data underlying the results available to ensure full reproducibility?

Yes

Is the study design appropriate and is the work technically sound?

Yes

Are the conclusions drawn adequately supported by the results?

Yes

Are sufficient details of methods and analysis provided to allow replication by others?

Yes

Reviewer Expertise:

Topology optimization

I confirm that I have read this submission and believe that I have an appropriate level of expertise to confirm that it is of an acceptable scientific standard, however I have significant reservations, as outlined above.

## References

[1] : Efficient topology optimization in MATLAB using 88 lines of code. *Structural and Multidisciplinary Optimization* .2011;43(1) : 10.1007/s00158-010-0594-7 1-16 10.1007/s00158-010-0594-7

# Response

**Published:** 2024-10-13

**Authors:** Marcio Cavalcante

The authors have many thanks to the reviewer for their valuable comments. The manuscript has been revised in accordance with the comments, and detailed revisions are listed below point by point.

*Comments to the Authors:*

**Reviewer comment: **The manuscript presents a Matlab implementation for topology optimization based on Finite Volume theory for 2D compliance problems.

 In general, the manuscript is interesting and is very useful for beginners of topology optimization. The Matlab code is fast, due to vectorization and memory pre-allocation, in a manner similar to the code top88 (Andreassen E, et al., 2011 [Ref 1]). Compared to the previous works by the same group of authors, computational time is reduced significantly. However, there are many unclear information that needs thorough revision by the authors.

 1) Although the approach may not require filter to avoid checkerboard pattern, it is clearly observed in the results that when no filter is applied, many thin components appear in the optimized design. Such thin components may be suffered from high stress values. In fact, as in the literature, filter also contributes to length-scale control. The authors should discuss this aspect.

***Author response:*** *The authors agreed with the reviewer, and the following texts were added to the manuscript:*

 Although the finite-volume theory employed in the TOP2DFVT algorithm effectively mitigates checkerboard patterns, filtering techniques remain essential for producing manufacturable designs. Filters such as sensitivity and density filters play a crucial role in controlling the characteristic length scale, thereby reducing the formation of thin structural elements that could cause fabrication and structural stability challenges. Additionally, applying these filters contributes to a smoother distribution of material, resulting in a more uniform stress distribution across the structure.

 However, results obtained without filters can also be explored effectively within the finite-volume theory framework. When working with coarser meshes, the larger subvolumes naturally limit the appearance of thin elements, which helps to define a more favorable topology from a manufacturing perspective. These larger subvolumes act as a resolution control mechanism, preventing the appearance of fine bars that could lead to structural issues such as localized buckling or stress concentration. Thus, the finite-volume theory provides a flexible approach for generating optimized topologies that balance manufacturability and structural robustness by carefully selecting the mesh size and employing or omitting filters as appropriate.

**Reviewer comment: **2) When there is no filter, it seems that clear “black and white” results could be obtained. Nevertheless, the authors should compute the non-discreteness values to quantitatively demonstrate this aspect.

 On the other hand, when filter (either sensitivity filter or density filter) is applied, gray elements appear. In order to reduce the gray elements and increase the discreteness of the solution, volume-preserving Heaviside projection is commonly employed. The authors should also mention this.

***Author response:*** *The authors agreed with the reviewer, and the following texts were added to the manuscript:*

 In topology optimization without filters, distinct "black and white" designs can typically be achieved, representing clear material and void regions. When filters such as sensitivity or density filters are applied, gray regions often emerge in the design. These gray elements represent intermediate density values that compromise the binary nature of the solution. To mitigate this issue and increase the discreteness of the topology, volume-preserving Heaviside projection is a commonly used strategy. The Heaviside projection function sharpens the transition between material and void by pushing intermediate densities towards 0 or 1, thereby reducing the gray regions. This technique preserves the total volume while enhancing the manufacturability of the optimized structure (Bendsøe and Sigmund 2003; Ferrari and Sigmund 2020). Future implementations of the proposed method could benefit from incorporating the Heaviside projection to refine the design’s discreteness further and improve its practical applicability in manufacturing processes, especially when employing filtering techniques.

# Reviewer report: Reviewer response for version 1

**DOI:** 10.5256/f1000research.165561.r306477

**Published:** 2024-08-12

**Authors:** Yingjun Wang

**Affiliations:** 1 South China University of Technology, Guangzhou, Guangdong, China

This paper presents a Matlab implementation for 2D topology optimization based on finite-volume method.  The Matlab code Top2DFVT uses the loop vectorization and memory preallocation of top88, which is much more efficient than the authors' previous work. Three examples are tested to demonstrate the validity and efficiency of Top2DFVT. However, there are still some comments for the authors.

 1. How to calculate the local stiffness matrix for a subvolume q  should be given.

 2. What is the meaning of the subscripts "pu", "pp", "uu", "up" in Eq.(13).

 3. In Section 3.1, there are two cases for the objective function gradient. Is only one of the cases in a subvolume, or the two cases can exist together in a subvolume?  

 4. In Section 4, an algorithm flowchart of Top2DFVT should be added. 

 5. In Top2DFVT, the memory preallocation accelerates the computational efficiency, but increases the memory usage. The authors should state this in the paper.

 6. For the illustrative example in Figure 5, why are the number of subvolumes in the horizontal and vertical directions set to 202 and 101, not 200 and 100?  

 7. In Tables 2-4, it can be found that the number of iterations of No filter cases is larger than the Sensitivity filter cases for the same mesh size, why? In addition, the number of iterations is more than 1000 when the number of elements increases, which is too many for the compliance minimization problem. The convergence history should be given for at least one example.

 8. The proposed method should be compared with finite-element-based topology optimization methods such as top88 or top99neo in detail to demonstrate its validity and efficiency.

Is the work clearly and accurately presented and does it cite the current literature?

Yes

If applicable, is the statistical analysis and its interpretation appropriate?

Yes

Are all the source data underlying the results available to ensure full reproducibility?

Partly

Is the study design appropriate and is the work technically sound?

Yes

Are the conclusions drawn adequately supported by the results?

Yes

Are sufficient details of methods and analysis provided to allow replication by others?

Yes

Reviewer Expertise:

topology optimization

I confirm that I have read this submission and believe that I have an appropriate level of expertise to confirm that it is of an acceptable scientific standard, however I have significant reservations, as outlined above.

# Response

**Published:** 2024-10-13

**Authors:** Marcio Cavalcante

The authors have many thanks to the reviewer for their valuable comments. The manuscript has been revised in accordance with the comments, and detailed revisions are listed below point by point.

***Comments to the Authors:***

 This paper presents a Matlab implementation for 2D topology optimization based on finite-volume method. The Matlab code Top2DFVT uses the loop vectorization and memory preallocation of top88, which is much more efficient than the authors' previous work. Three examples are tested to demonstrate the validity and efficiency of Top2DFVT. However, there are still some comments for the authors.

**1) How to calculate the local stiffness matrix for a subvolume q should be given.**

***Response: As mentioned in the manuscript, the local stiffness matrix calculation can be found in Araujo et al. (2020a).*** **2)**

**2) What is the meaning of the subscripts "pu", "pp", "uu", "up" in Eq.(13).**

***Response: The following description of the submatrices in Eq. (13) was incorporated into the text:***

 The subscripts in Eq. (13) reflect the relationship between prescribed and unknown quantities, where the first index refers to forces and the second to displacements. The letter "u" denotes unknown quantities, while "p" represents prescribed ones. Therefore, the submatrix Kpu expresses the coupling between prescribed forces and unknown displacements, while Kpp defines the relationship between prescribed forces and prescribed displacements. Similarly, Kup​ describes the interaction between unknown forces and prescribed displacements, and Kuu corresponds to the relationship between unknown forces and unknown displacements. This indexing scheme distinguishes between the interactions of prescribed and unknown quantities within the stiffness matrix.

**3) In Section 3.1, there are two cases for the objective function gradient. Is only one of the cases in a subvolume, or the two cases can exist together in a subvolume?**

***Response: The two cases mentioned in section 3.1 refer to the global system of equations that relates the global force vector with the global displacement vector. The following text was added to the manuscript to clarify the case used in the analyzed examples:***

 The examples analyzed in the following sections are associated with the second case, where a prescribed force is applied, and all the prescribed displacements are zero.

**4) In Section 4, an algorithm flowchart of Top2DFVT should be added.**

***Response: The authors agreed with the reviewer, and the following flowchart was added to the manuscript.***

****

**Figure 4. **Flowchart of the Top2DFVT for the topology optimization of linear elastic continuum structures.

**5) In Top2DFVT, the memory preallocation accelerates the computational efficiency, but increases the memory usage. The authors should state this in the paper.**

***Response: The authors agreed with the reviewer, and the following text was added to the manuscript:***

 In the Top2DFVT implementation, memory preallocation is crucial in enhancing computational efficiency by reducing the time required for matrix assembly and solving linear systems. However, this performance gain comes at the expense of increased memory usage, as large data structures must be allocated in advance. This trade-off between computational speed and memory consumption is a common consideration in the design of topology optimization algorithms. While the memory overhead is manageable for medium-sized problems, it can become significant for large-scale applications. Therefore, careful management of memory resources is essential to balance efficiency and scalability.

**6) For the illustrative example in Figure 5, why are the number of subvolumes in the horizontal and vertical directions set to 202 and 101, not 200 and 100?**

***Response:* *The following text was added to the manuscript:***

 In this example, the dimensions of the cantilever deep beam are L=100 and H=50, and the concentrated load is applied at the center of the free edge. To ensure the load is applied to the face of a single subvolume, the number of subvolumes in the vertical direction must be odd. Furthermore, to generate an analysis domain with square subvolumes, the number of subvolumes in the horizontal direction must be twice the number of those in the vertical direction.

**7) In Tables 2-4, it can be found that the number of iterations of No filter cases is larger than the Sensitivity filter cases for the same mesh size, why? In addition, the number of iterations is more than 1000 when the number of elements increases, which is too many for the compliance minimization problem. The convergence history should be given for at least one example.**

***Response:* *The following text and figure were added to the manuscript:***

 Adopting the continued penalization scheme, combined with a highly restrictive local convergence criterion, increases the number of iterations in the investigated examples, especially for the most refined meshes, where achieving local convergence is more challenging. The smooth local response produced by the sensitivity filter accelerates convergence compared to the optimization process without filtering techniques, a result not observed with the density filter. Figure 9 displays the objective function histories throughout the optimization process of the cantilever deep beam for the SIMP and RAMP methods, highlighting that the density filter demands more iterations for convergence. In contrast, the optimization process with the sensitivity filter achieves the fastest convergence. The RAMP method provides closer values for the objective function throughout the optimization process for the three approaches adopted: results without filters, with the sensitivity filter, and with the density filter. Furthermore, the RAMP method demonstrates a more stable convergence process than the SIMP method for these approaches. Notably, the jumps in the continued penalization scheme are significantly smaller for the RAMP method than those between different penalty factors in the SIMP method.

****

**Figure 9.** Objective function histories for the SIMP and RAMP methods.

**8) The proposed method should be compared with finite-element-based topology optimization methods such as top88 or top99neo in detail to demonstrate its validity and efficiency.**

***Response:* *The authors agreed with the reviewer, and the following texts and figures were added to the manuscript:***

 The algorithm known as top99neo, proposed by Ferrari and Sigmund in 2020, is used as a benchmark source for the traditional finite-element method for bilinear elements. The algorithm uses the continued penalization scheme, gradually increasing the penalty factor (∆p=0.25) from 1 to 4, similar to the finite-volume theory approach. Each penalty factor is subjected to 25 iterations. The Heaviside projection parameters are updated throughout the optimization process. The parameter β starts at 2 and increases to 16 by an increment of ∆β=2 every 25 iterations. Figure 12 shows the optimized topologies obtained using the density filter. The filter radius changes to reflect the exact size of the filter radius employed in the finite-volume theory approaches for each mesh size (1.5, 3, and 6, respectively). The optimized topologies obtained using the finite-element approach are more mesh-dependent, even when filtering strategies are employed, as compared to the optimized topologies obtained using the finite-volume theory approach, especially in the case of the results with the sensitivity filter. The computational cost for the finest mesh is 2 minutes and 30 seconds, with a maximum of 350 iterations. The total number of degrees of freedom for the finite-element approach is 132132, while the finite-volume theory approach has a total of 260280 degrees of freedom, which partially explains the difference in computational costs. As the top99neo algorithm employs finite elements with unitary dimensions, it is not feasible to compare the obtained values for the objective function of the optimized topologies.

****

**Figure 12.** Optimized topologies for the cantilever deep beam employing the top99neo algorithm.

 Figure 16 shows the optimized topologies obtained employing the top99neo algorithm, where the penalty factor increases gradually (∆p=0.25) from 1 to 4 after every 25 iterations until a maximum of 350 iterations, and the beta parameter increases gradually from 2 to 16 (∆β=2), after every 25 iterations, similarly to the cantilever deep beam example. The optimized topologies shown in Figure 16 are mesh dependents even when filtering strategies are employed. The filter radius is assumed to be 1.5 for the 90x30 mesh, 3 for the 180x60 mesh, and 6 for the 360x120 mesh. When a similar filter radius is employed in the context of the finite-volume theory, this technique demonstrates a less mesh sensitivity behavior when compared to finite element-based strategies. Regarding computational efficiency, the top99neo code has presented a processing time of 86 seconds for the finest mesh. Generally, the Top2DFVT algorithm presents a higher computational cost when compared to the Q4 finite element-based algorithm. The number of degrees of freedom partially explains this higher computational cost once the total number of degrees of freedom for the finest mesh considering the top99neo algorithm is 87362, while in the Top2DFVT algorithm, the total number of degrees of freedom for the same analysis is 173760. Furthermore, the number of iterations for this example is relatively high for the finite-volume theory approaches once the top99neo algorithm adopts a maximum of 350 iterations.

****

**Figure 16.** Optimized topologies for the MBB beam employing the top99neo algorithm.

 The top99neo algorithm is also performed considering the L-bracket beam problem, and the obtained optimized topologies can be observed in Figure 20. As in the previous examples, the same numerical parameters are employed for the physical model, such as the gradual increase in the penalty factor, from 1 to 4 with ∆p=0.25, and in the Heaviside Projection β parameter, from 2 to 16 with ∆β=2. In terms of computational cost, the top99neo code has presented a processing time of 1 minute and 1 second for the finest mesh, considering a total of 350 iterations, while the Top2DFVT algorithm has presented a processing time of 39 seconds with a total of 155 iterations for of the SIMP method and the sensitivity filter. This difference in processing time can be partially explained by the difference in the total number of iterations observed during the topology optimization analyses. For this example, the Top2DFVT algorithm, considering the sensitivity filter approach, presents a reduced number of iterations for convergence.

****

**Figure 20.** Optimized topologies for the L-bracket beam employing the top99neo algorithm.
